# Applications of Raman spectroscopy in cancer diagnosis

**DOI:** 10.1007/s10555-018-9770-9

**Published:** 2018-12-19

**Authors:** Gregory W. Auner, S. Kiran Koya, Changhe Huang, Brandy Broadbent, Micaela Trexler, Zachary Auner, Angela Elias, Katlyn Curtin Mehne, Michelle A. Brusatori

**Affiliations:** 1 Michael and Marian Ilitch Department of Surgery, School of Medicine, Wayne State University, 5050 Anthony Wayne Drive, Detroit, MI 48202 USA; 2 Department of Biomedical Engineering, College of Engineering, Wayne State University, 5050 Anthony Wayne Drive, Detroit, MI 48202 USA; 3 Smart Sensors and Integrated Microsystems Program, Wayne State University, Detroit, MI 48202 USA; 4 Henry Ford Health Systems, Detroit Institute of Ophthalmology, Grosse Pointe Park, MI 48230 USA; 5 Department of Physics & Astronomy, Wayne State University, Detroit, MI 48202 USA

**Keywords:** Raman spectroscopy, Applications, Clinical, Cancer, Diagnosis, Spectroscopy

## Abstract

Novel approaches toward understanding the evolution of disease can lead to the discovery of biomarkers that will enable better management of disease progression and improve prognostic evaluation. Raman spectroscopy is a promising investigative and diagnostic tool that can assist in uncovering the molecular basis of disease and provide objective, quantifiable molecular information for diagnosis and treatment evaluation. This technique probes molecular vibrations/rotations associated with chemical bonds in a sample to obtain information on molecular structure, composition, and intermolecular interactions. Raman scattering occurs when light interacts with a molecular vibration/rotation and a change in polarizability takes place during molecular motion. This results in light being scattered at an optical frequency shifted (up or down) from the incident light. By monitoring the intensity profile of the inelastically scattered light as a function of frequency, the unique spectroscopic fingerprint of a tissue sample is obtained. Since each sample has a unique composition, the spectroscopic profile arising from Raman-active functional groups of nucleic acids, proteins, lipids, and carbohydrates allows for the evaluation, characterization, and discrimination of tissue type. This review provides an overview of the theory of Raman spectroscopy, instrumentation used for measurement, and variation of Raman spectroscopic techniques for clinical applications in cancer, including detection of brain, ovarian, breast, prostate, and pancreatic cancers and circulating tumor cells.

## Introduction

Noninvasive or minimally invasive *in vivo* tools that can provide rapid tissue assessment and/or monitor treatment therapies have potential application in many fields of medicine. Interest in clinical spectroscopy is rising due to the potential of vibrational spectroscopic techniques for noninvasive tissue diagnostics. Spectroscopic techniques involve the study of the interaction matter with light. Molecules are composed of two or more bonded atoms that are in continuous motion (be it electronic, vibrational, rotational, or translational). Due to the different kinds of motion and intermolecular interactions, a molecule possesses different forms of energy that can be probed with electromagnetic radiation to obtain information on molecular structure and composition. A molecule can react to incoming light *via* the processes of absorption and scattering. The process of absorption occurs when a material takes up radiant energy internally. Since energy is quantized, there are distinct energy levels in a molecule that correspond to different amounts of rotational, vibrational, and electronic energy. If the energy of a photon matches a difference between two energy levels in a molecule, absorption can occur causing a transition from the lower to higher energy state. Rotational transitions occur at low energies (microwave region of the electromagnetic spectrum), while vibrational transitions occur in the infrared (IR), and electronic transitions occur in the visible and ultraviolet (UV) region of the electromagnetic spectrum [1].

Conversely, scattering can occur without an energy level transition. When light, with insufficient energy to cause excitation, impinges on a molecule, it can be scattered at the same frequency as the incident light [2]. This is termed elastic scattering and is typically describe by Rayleigh or Mie theory. While most light is elastically scattered from molecules, some light can be scattered at frequencies that differ from the incident radiation and is termed inelastic scattering. Unlike the elastic process, inelastic scattering involves a net energy transfer between the incident photons and a material [2]. Fluorescence and Raman scattering are examples of inelastic processes.

Both Raman and infrared (IR) spectroscopy probe molecular vibrations associated with chemical bonds in a sample to obtain information on molecular structure, composition, and intermolecular interactions. IR spectroscopy and Raman spectroscopy are complementary techniques that differ in their methodology to probe vibration. IR spectroscopy monitors the net absorption of incident radiation by a sample in the IR region of the electromagnetic spectrum (and depends on a net change in dipole moment of a molecule as it vibrates/rotates). The wavelength of IR absorption bands is characteristic of vibrational modes of specific bond types in a sample, whereas Raman spectroscopy profiles vibrational and rotational motion of molecules that arise from an inelastic scattering event that depends on nuclear vibrations that create a change in polarizability of a molecule as it vibrates/rotates. Thus, Raman spectroscopy provides intensity profiles of scattered light as a function of frequency. The frequency difference between the incident and scattered light is the frequency of vibration. The vibrational frequency at which Raman bands occur is characteristic of vibrational modes of specific bond types in a molecule, with the intensity directly proportional to the concentration of molecular constituents that give rise to the bands. Vibrations that are Raman active may not be IR active, and *vice versa*, or they may be strong in one effect and weak in the other. Due to strong water absorbance in the IR region of the electromagnetic spectrum, analysis of aqueous solutions or tissue with high water content may be difficult with IR spectroscopy, whereas the Raman water signal is weak making it an ideal technique for *in vivo* tissue interrogation. Since Raman spectroscopy is a nondestructive, reagentless, vibrational spectroscopic technique, it provides rapid molecular characterization of tissue *in vivo* or *in vitro* for biopsy, margin assessment, therapeutic evaluation, or laboratory use. The spectroscopic profile arising from the unique composition of Raman-active functional groups of nucleic acids, proteins, lipids, and carbohydrates that each sample has allows for the evaluation, characterization, and discrimination of tissue type. Numerous experimental studies have demonstrated the capability of Raman spectroscopy for tissue characterization in neurosurgical application and for evaluation of breast, prostate, ovarian, and pancreatic cancers, among others. This review summarizes some of the Raman work to date for pathophysiological evaluation of cancerous tissue, for characterizing circulating tumor cells to determine their relation to the primary tumor and the metastasis process, and discusses the future of Raman spectroscopy for clinical oncology applications.

## Theory of Raman spectroscopy (spontaneous Raman scattering)

### Classical theory

The Raman effect was discovered in 1928 by CV Raman when he observed that light traveling through various liquids scatter differently in a behavior distinct from fluorescence [3]. This inelastic molecular vibration/rotation phenomenon that causes a change in the polarizability of a molecule occurs in approximately 1 in 10^7^ photon interactions with matter [4]. The polarizability of a molecule represents the ability of an external electric field, of strength *E*, to induce a dipole moment, *μ*_ind_ (or an additional dipole moment), in the molecule. For a small field, the induced dipole moment can be expressed as [5, 6]:

1 $$ {\upmu}_{\mathrm{ind}}=\upalpha E $$

and the electric field of the incident light by [5, 6]:

2 $$ E={E}_0\cos \left(2\uppi {t\upnu}_0\right) $$

where *E*_0_ is the field strength and *ν*_0_ is the frequency of oscillation. For any molecular bond, the individual atoms in a molecule are confined to specific vibrational modes. The displacement, *Q*, of atoms about their equilibrium position due to a particular vibrational mode can be defined by [5, 6]:

3 $$ Q={Q}_0\cos \left(2\uppi {t\upnu}_{\mathrm{v}}\right) $$

where *Q*_0_ is the amplitude and *ν*_v_ is the frequency of vibration. For small displacements (such as that of a typical diatomic molecule), polarizability can be approximated as a Taylor series expansion in normal coordinates [5, 6]:

4 $$ \upalpha ={\upalpha}_0+{\left(\frac{\mathrm{\partial \upalpha }}{\partial Q}\right)}_0Q $$

The polarizability has a static term and a sinusoidal oscillating term. For Raman scattering to occur, the polarizability needs to change with vibration, ${\left(\frac{\mathrm{\partial \upalpha }}{\partial Q}\right)}_0\ne 0$. Here, the subscript 0 indicates that the parameters *α*_0_ and (∂*α*/∂*Q*)_0_ are evaluated at the equilibrium position of the atoms. Substituting Eqs. 2, 3, and 4 into Eq. 1 yields [5, 6]:

5 $$ {\mu}_{\mathrm{ind}}={\alpha}_0{E}_0\cos \left(2\pi t{v}_0\right)+{\left(\frac{\partial \alpha }{\partial Q}\right)}_0\frac{E_0{Q}_0}{2}\left[\cos \left(2\pi \left({v}_0+{v}_{\mathrm{v}}\right)t\right)+\cos \left(2\pi \left({v}_0-{v}_{\mathrm{v}}\right)t\right)\right] $$

Classically, an oscillating induced dipole moment emits radiation at the frequency of oscillation. The first term of the equation represents an oscillating dipole that emits radiation at the same frequency, ν_0_, of the incident light (Rayleigh scattering). The second term of the equation represents Raman scattering. The oscillating polarizability causes an induced dipole moment that oscillates and emits radiation at frequencies (*ν*_0_ ± *ν*_v_) that differ from the incident light. The classical picture cannot account for many aspects of Raman scattering observed experimentally, such as the intensities of scattered light. Quantum mechanical treatment can provide a more detailed description that involves quantized energy levels and wave functions of the molecule.

### Quantum description

**Fig. 1:**
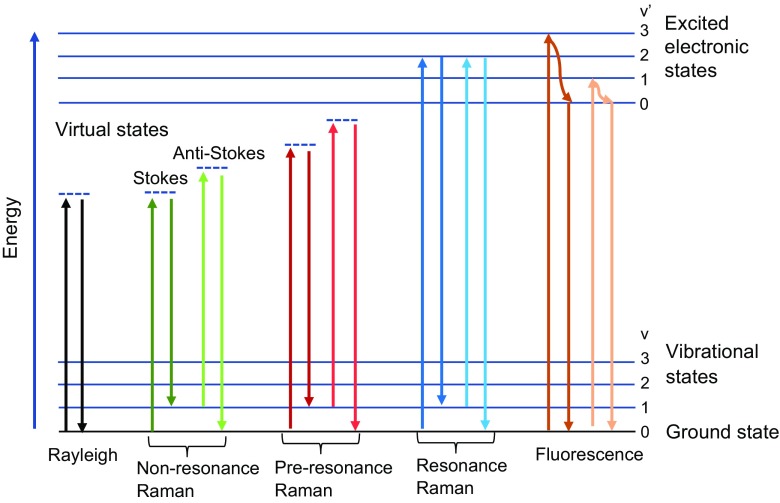
Energy level diagram for Rayleigh scattering, Raman scattering, and fluorescence

Atoms joined by bonds are confined spatially resulting in molecular vibrations/rotations to occur at discrete energy levels. For a diatomic molecule undergoing simple harmonic motion (vibration), energy can be written as:

6 $$ {\mathrm{E}}_j=\left(j+\frac{1\ }{2}\right)h{\upnu}_{\mathrm{v}}\kern1em \mathrm{for}\ j=1,2,3\dots $$

where *ν*_v_ is the frequency of a vibrational mode, *h* is Plank’s constant, and *j* is the quantum number. A simple energy level diagram is shown in Fig. 1. As illustrated, when light is incident on a molecule at an initial ground state, *j* = 0, with energy *E*_0_, it can be prompted to a virtual energy level (very short-lived, unobservable quantum state) and quickly return to the initial state. Since there is no energy level transition, photons are emitted at the same energy (frequency) as the incident light (Rayleigh scattering). Raman scattering also involves an intermediate virtual energy state. In this case, there is an energy transfer between the incident light and molecule. The Raman emission occurs as two possible outcomes, Stokes or anti-Stokes scattering. Stokes scattering occurs when a molecule is initially in the ground state, *j* = 0, with energy *E*_0_ = (1/2) *hν*_v_ and is transitioned to a virtual energy level and then relaxes to an excited state, *j* = 1, with energy *E*_1_ = (3/2) *hν*_v_ [7]:

7 $$ {E}_1=\mathrm{final}\ \mathrm{energy}\ \mathrm{state}\ \mathrm{of}\ \mathrm{molecule}={E}_0+h{\upnu}_{\mathrm{v}} $$

A Raman mode is active only if the polarizability changes during a molecular motion (vibration/rotation). In the quantum description, a transition electric dipole and polarizability replaces the oscillating electric dipole and polarizability. The transition moment leads to a transition between two quantum states, *i* and *f*, only if it is nonzero [8]:

8 $$ {M}_{\mathrm{ind}}\left(i\to f\right)={\left(\frac{\mathrm{\partial \upalpha }}{\partial Q}\right)}_0{\mathcal{E}}_0\int {\uppsi}_iQ\ {\uppsi}_f\ \mathrm{d}Q\ne 0 $$

where *M*_ind_ is the Raman transition moment for a diatomic molecule, *ψ*_*i*_ and *ψ*_*f*_ are wave functions (solutions to the time-dependent Schrodinger equation) for states *i* and *f*, *α* is the polarizability operator (tensor property), 퓔_0_ is the amplitude of the electric field, and *Q* are the coordinates. Since energy is conserved, the gain in energy, *hν*_v_, by the molecule results in an equal amount of energy, *hν*, being removed from the incident photon, where *hν*_0_ is incident energy. This change in energy is the energy of a scattered photon [7]:

9 $$ \mathrm{energy}\ \mathrm{of}\ \mathrm{a}\ \mathrm{scattered}\ \mathrm{photon}=h\left({\upnu}_0-\upnu \right)=h\left({\upnu}_0-{\upnu}_{\mathrm{v}}\right) $$

Since wavelength is inversely proportional to the frequency, radiation is emitted at longer wavelengths (lower energy) than the incident light.

As depicted in Fig. 1, anti-Stokes scattering occurs when a molecule is initially in an excited state prior to irradiation with *E*_1_ = (3/2) *hν*_v_ and is promoted to a virtual energy level, then relaxes to the ground state with *E*_0_ = (1/2) *hν*_v_ after scattering [7]:

10 $$ {E}_0=\mathrm{final}\ \mathrm{energy}\ \mathrm{state}\ \mathrm{of}\ \mathrm{molecule}={E}_1-h{\upnu}_{\mathrm{v}} $$

Here, energy is removed from the molecule. This corresponds to photon energy, *hν* = *hν*_v_, being transferred to the energy of the incident photon. The energy of a scattered photon is expressed by Eq. 10 [7]:

11 $$ \mathrm{energy}\ \mathrm{of}\ \mathrm{a}\ \mathrm{scattered}\ \mathrm{photon}=h\left({\upnu}_0+{\upnu}_{\mathrm{v}}\right) $$

Radiation is emitted at shorter wavelengths (higher energy) than the original radiation. However, since Stokes-shifted scatter is more intense than anti-Stokes, Stokes scatter is typically measured.

With conventional Raman spectroscopy, the effect is independent of wavelength since no real energy states are involved (only virtual states). This is termed nonresonance Raman. Certain substances, when exposed to electromagnetic radiation, can produce a strong fluorescence signal that overlaps the Raman signal. Raman scattering and fluorescence are competing phenomena that have similar origin. With the Raman effect, molecules are excited to a virtual energy level for a short period, on the order of picoseconds, before a photon is emitted. Whereas in fluorescence, incident light is absorbed by a molecule and re-emitted from electronically excited states after a resonance time on the order of nanoseconds. Here, light is typically emitted at a longer wavelength than the incident light.

In contrast, resonance Raman spectroscopy, a variant of conventional Raman, measures molecular vibrations in a wavelength-dependent manner. When the wavelength of the exciting source coincides with an electronic transition of the molecule, a resonance effect is observed and the intensity of some Raman-active vibrations can be increased by a factor of 10^2^–10^6^.

## Instrumentation and Raman spectra

### Laboratory instrument

Raman instruments built for laboratory research are typically used in *ex vivo* applications. Such systems, geared toward research and development studies, are typically constructed to collect high-quality spectra with the ability to use different excitation/detection wavelengths and data acquisition times to determine and refine experimental parameters. These systems are also used to develop and test statistical algorithms/models for material/tissue characterization. For tissue interrogation, the Raman spectra can be obtained at discrete points or from an area by mapping. With spatial mapping, the laser spot scans the sample at preset steps and a Raman spectrum is obtained at each point. This technique can be used to render 1-D profiles, 2-D images, or 3-D volumes. Variation in spectral information from different points on the sample can be obtained using the intensity of a particular Raman band or by utilizing the entire spectra. Raman imaging techniques allow visualization and quantification of the distribution of different components in an area of the sample.

**Fig. 2:**
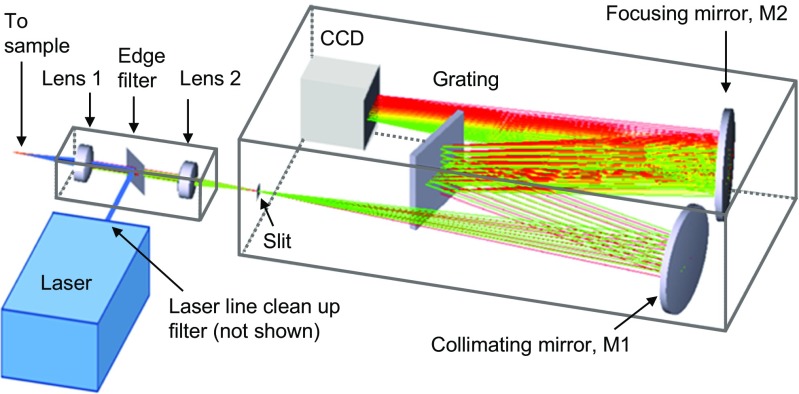
Typical laboratory Raman spectrometer

Figure 2 shows the configuration of a typical laboratory Raman system. Light from a laser is reflected off a long pass edge filter (or notch filter) and is directed through lens 1 that focuses the laser light onto the sample. Light scattered off the sample is collected in a 180° backscatter geometry. Light collected by lens 1 is directed to the edge filter which blocks the laser light and lets only the Raman scattered light through. The Raman scattered light is focused by lens 2 onto the entrance slit of the spectrometer. Light entering through the slit is collimated by mirror M1 and directed onto the grating of the spectrometer. The grating disperses the light focused by mirror M2 into images of the entrance slit on the charge-coupled device (CCD).

### Raman probe

A small footprint high-resolution system that enables rapid measurement is desired for *in vivo* clinical application. Typically, Raman fiber optic probe is employed to allow access to organs. The smaller footprint can translate to lower resolution or smaller spectral range of measurement compared to a laboratory research-grade instrument. Lower resolution means information may be lost and spectral features may not be differentiated. Shorter measurement times can translate to a lower signal-to-noise ratio. However, advancements in instrumentation that improve sensitivity while reducing size and cost and strategies to promote signal enhancement are under development that will increase the feasibility of Raman spectroscopy for clinical use.

**Fig. 3:**
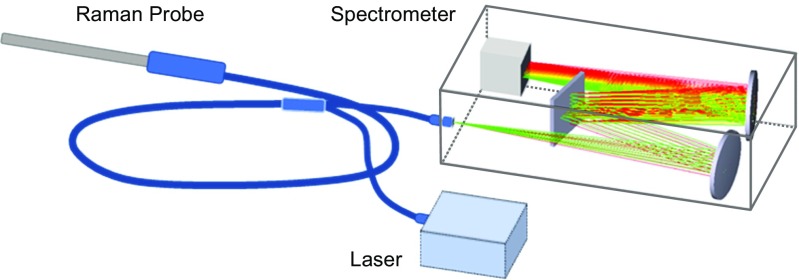
Raman probe assembly

Figure 3 shows a schematic of a Raman probe. Light from the laser transverses through an optical fiber and through a laser line cleanup filter that is internal to the probe. This filter suppresses unwanted signals including those that can arise from the fiber itself. The laser light is then focused onto the sample with an internal lens (or assembly of lenses). Backscattered light is collected *via* the lens and directed through an edge filter (internal in the probe) that allows only the Raman signal to pass though. The Raman scattered light is then coupled into a second fiber or assumedly of fibers that connect to the spectrometer at the slit.

### The Raman spectra

A Raman spectrum is obtained by plotting the intensity of scattered light as a function of frequency. By convention, the frequency of scattered light is converted to Raman shifts, the difference in frequency between the incident and scattered light (usually in units of wavenumbers (cm^−1^)). Because energy levels are quantized, Raman scattering occurs at discrete wavelengths that correspond to the energy level transition. Since each type of sample has a distinctive chemical composition and molecular structure, a characteristic spectral fingerprint of the sample is obtained.

**Fig. 4:**
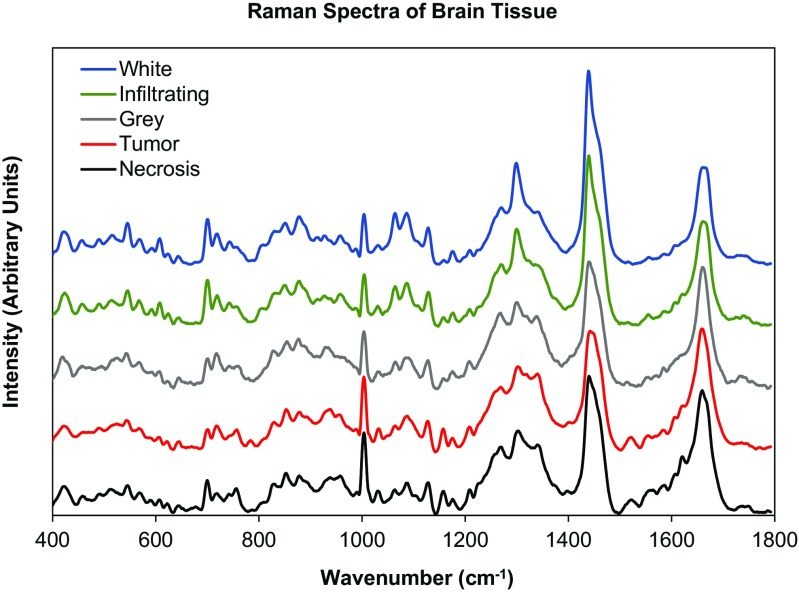
Mean Raman spectra of interoperative brain tissue samples in the spectral range of 400–1800 cm^−1^ deemed as normal, white matter, gray matter, tumor (GBM), infiltrating tumor, and necrosis

Figure 4 shows the Raman spectra of surgically excised brain tissue in the spectral region of 400–1800 cm^−1^ deemed by histopathology as normal (gray matter and white matter), tumor (GBM), infiltrating tumor, and necrosis. It is evident that the spectroscopic profile of each tissue type is unique and can provide a basis for characterization and differentiation.

Raman spectra are complex in nature and often contain broad peaks due to an ensemble effect with contributions arising from all the molecules present in the sample. Characteristic Raman peaks that correspond to the vibration/rotation of functional groups of atoms in the Fig. 4 sample are as follows: (1) in the region between 1760 and 1500 cm^−1^ arise from C=O stretching vibrations (amide I band) with contributions of water, proteins (C=C), nucleic acids, and lipids (C=C stretch); (2) bands in the region between 1500 and 1400 cm^−1^ are due to C–H, CH_2_, and CH_3_ vibrations; and (3) in the region between 1400 and 1200 cm^−1^ arise from C–N stretching and N–H bending (amide III band) with contributions from proteins (CH_3_CH_2_ wagging, twisting, bending), polysaccharides, lipids (CH_3_CH_2_ twisting, wagging, bending), and nucleic acids. (4) The region between 1200 and 800 cm^−1^ has contributions from nucleic acids, lipids (C–C, C–O stretching), proteins (C–C, C–N stretching), and C–O stretching of carbohydrates, and (5) the region between 800 and 600 cm^−1^ has vibrations associated with nucleotide conformation, cholesterol, and phosphodiesters. The peak location of an isolated functional group is typically known. However, the actual peak location of a functional group in a molecule may differ (shift) from the isolated case because of interactions and bonding with its neighbors [9].

### Analysis of spectroscopic data

Continued advancements in analysis methodologies are paramount in biomedical Raman spectroscopy. There are a variety of methods used to correlate Raman spectroscopic data with tissue type for diagnostic evaluation. Methods that use discrete Raman bands to distinguish and discriminate between tissue types have been widely used to develop statistical models or classification algorithms. Alternatively, whole spectrum analyses using machine learning techniques are also used for tissue discrimination. Whether discrete bands or whole spectra are used to develop automated tissue classification schemes, algorithms need to be robust and have low classification error. There are several factors that can affect the result of many analysis methods such as spectral preprocessing. Various interferents can hamper the interpretation of Raman spectra of biological samples such as fluorescence or other additive features that contribute to the baseline noise in the raw spectra. Preprocessing the raw data helps eliminate unwanted signals and enhance Raman spectral features. Two basic preprocessing steps are typically required to achieve reproducible qualitative and quantitative data: (1) baseline corrections to remove spectral contributions due to fluorescence and (2) a normalization procedure to remove focusing effects and effects that can arise from laser intensity fluctuations. Since preprocessed spectra are typically analyzed, these methodologies are important to consider.

## Variations of Raman spectroscopy

Since spontaneous Raman scattering is weak, many techniques have been developed to improve the signal-to-noise ratio. Table 1 identifies several of these methods that deviate from standard Raman spectroscopy and include the challenges to incorporating them into a diagnostic or intraoperative surgical tool.

**Table 1:** Variations of Raman spectroscopy [10–27]

Surface enhanced Raman spectroscopy (SERS)	
Method	SERS employs metallic nanostructures, typically gold, silver, or platinum as a substrate. Electromagnetic enhancement is considered the dominant contributor to most SERS processes [10, 11]. This involves the interaction of surface plasmons (generated by incident light) on metallic nanostructures with Raman-active molecules. Light from a laser beam excites surface plasmons (collective oscillations of conduction electrons) in the metal. Resonant interaction between the incoming laser light and surface plasmons leads to an enhanced electric field (whose magnitude may be many orders stronger than that of the incident light) in areas around the metallic nanostructures. The enhanced field created at the surface of the nanostructures is localized to a region of a few nanometers from the surface. Molecules nearby or absorbed on the metallic substrate experience the enhanced field, which subsequently can lead to an order of magnitude increase in signal strength of Raman scattered light [10, 12].
Advantage over standard Raman	Significant enhancement of Raman signal is reported by a factor of 10^5^ to 10^10^.
Disadvantage for intraoperative use	Requires additional steps during surgery such as adding a nanoprobe molecule to the tissue of interest for enhancement [10], SERS tag must be biocompatible, and Raman measurement must be in close proximity to tag. The analysis is confined to tens of nanometers from the nanoparticle or probe. The variability of the nanoparticles creates nonreproducible results.
Tip enhanced Raman spectroscopy (TERS)	
Method	TERS achieves an analogous signal enhancement by focusing incident light onto a nanometer-scale metal coated tip of a scanning probe microscopy (SPM) cantilever. Tips are typically a Si or Si_3_N_4_ base coated by a thin evaporation deposit of Ag or Au [13], though protected TiN_*x*_ tips have shown potential as a cheaper and more durable alternative for aqueous solutions [14] The gap distance between the tip and the substrate is precisely regulated by the SPM controller allowing for subnanometer spatial resolution. Various SPM technologies, including atomic force microscopy (AFM), scanning tunneling microscopy (STM), and scanning nearfield optical microscopy (SNOM) [13, 15], may be used concurrently with an inverted Raman spectroscopy geometry to obtain coincident SERS spectra and SPM images [13]. TERS provides a unique opportunity to assign spectral characteristics to topographical locations and correlate to mechanical properties.
Advantage over standard Raman	As with SERS, significant enhancement of Raman signal is reported by a factor of 10^10^ [13]. Raman spectra obtained may be mapped to the substrate SPM image to facilitate identification of spectral peaks.
Disadvantage for intraoperative use	Due to its inverted optical geometry, traditional TERS techniques require the incident light to be focused though the bottom of the substrate onto the point of the SPM tip. Consequently, these techniques can only interrogate nearly clear or extremely thin substrates. To address this, side, top, and parabolic illumination configurations have been developed to interrogate opaque samples from above without disturbing the SPM functionality. However, these alternative geometries require excitation illumination to be applied off-axis creating an elliptical focal spot. This induces a larger focal surface area and stronger far field background noise resulting in lower SNR compared to traditional bottom illumination [17]. SPM also requires robust sample preparation and data interpretation sensitive to ambient conditions making it unsuitable for the surgical suite [16].
Resonance Raman scattering (RRS)	
Method	Signal enhancement with resonance Raman is achieved when the frequency of incident radiation coincides with the frequency of an electronic transition of a molecule. This provides energy to excite electrons to a higher electronic state. This technique can selectively augment signals affiliated with chromophores and other large conjugated molecules. Even in a complex sample with numerous vibrational modes, RR spectroscopy allows one to look at relatively few vibrational modes at a time. This can reduce the complexity of the spectrum to allow for easier identification. However, RRS often suffers from fluorescence background, which can obscure the Raman signals but may be avoided using short (deep UV) wavelengths [18].
Advantage over standard Raman	Increased signal strength is reported by a factor of 10^2^ to 10^6^.
Disadvantage for intraoperative use	RRS provides more limited/selective molecular information. Nonresonance-enhanced bands may seemingly disappear under the intensity of resonance-enhanced spectral peaks. Requires a tunable laser to selectively isolate the contributions from different chromophores. Carotenoids show enhancement in the visible region of the spectra, while DNA is enhanced in the UV region. UV laser sources can cause cellular damage. Fluorescence backgrounds can be significant due to excitation coinciding with UV–visible absorption [18].
Surface enhanced resonance Raman scattering (SERRS)	
Method	SERRS signal enhancement is due to a combined effect of SERS and RRS. SERS is achieved when molecules are in contact (or in close vicinity) with nanostructures that support surface plasmon resonance (SPR). The Raman signal is enhanced due to an increase of the EM field at/near the surface of the nanostructures due to the interaction of light with the substrate. The Raman signal is further amplified by tuning the excitation source (laser) to match an internal electronic transition of the adsorbed molecule [19].
Advantage over standard Raman	Increased signal strength is reported by a factor 10^13^ and 10^15^ [19].
Disadvantage for intraoperative use	Nanoprobe molecules (SERS nanoparticle) need to be added the tissue of interest [10]. For *in vivo* application, the SERS tags must be biocompatible. Another disadvantage is that only materials in close proximity to the tag will be subjected to measurement. The variability of the nanoparticles creates nonreproducible results. Resonance enhancement provides limited/selective molecular information. This feature has limited benefit depending on the application.
Spatially offset Raman spectroscopy (SORS)	
Method	Traditional Raman spectral acquisition of tissue is typically obtained using a 180° backscatter geometry and is limited to near-surface measurements within the first few hundred microns of the surface. Spatially offset Raman spectroscopy (SORS) enables measurements from subsurface layers in diffusely scattering media [20] and information as deep as 4 mm into the sample [21]. As opposed to traditional Raman, where laser illumination and collection are from the same area of the sample, SORS involves collecting the scattered light from a point that is laterally offset from the laser illumination. For a two-layered sample, two measurements are required to recover the Raman spectra of the individual layers. One spectrum is typically taken at zero offset, while the other is taken at nonzero offset. For this case, a scaled subtraction of the two spectra may be sufficient to recover the spectrum of the sublayer. For a multilayered system, more sophisticated methods may need to be employed. Clinical applications of this technique can be extended to bone [21] and breast tumor margin evaluation [22].
Advantage over standard Raman	Using an offset collection point allows data to be collected from deeper within the area of interest, up to 4 mm was demonstrated [21]. By comparison, standard Raman only penetrates a few hundred micrometers. Reduces tissue fluorescence.
Disadvantage for intraoperative use	Interrogation and collection offset of at least 3.5 mm are recommended, tumor thickness detection limitation of 2 mm (breast tissue) [22]; complex hardware requirements.
Transmission Raman spectroscopy (TRS)	
Method	TRS is considered a form of SORS, with collection and illumination points being on opposite sides of the sample. Unlike SORS, it is unable to provide the signatures of individual layers within the sample. Instead, it provides information on the entire sample volume.
Advantage over standard Raman	Unlike standard Raman spectroscopy, TRS has the potential to collect data from deeper within an area of interest.
Disadvantage for intraoperative use	For tissue interrogation, the coupling of laser radiation into deep tissue layers is hindered by losses of laser radiation at the surface of the sample from scattering as well as the diffuse nature of photon propagation through tissue [23]. However, by employing a dielectric filter on the surface of the tissue, Stone et al. detected Raman signals from depths of up to 2.7 cm within a breast phantom made up of porcine tissues [23].
Coherent anti-Stokes Raman scattering (CARS)	
Method	CARS is a 3rd-order nonlinear process that typically employs picosecond pulsed lasers. With this technique, a pump laser at a frequency *ω*_p_, a probe at a frequency *ω*_pr_, and a Stokes laser at a frequency *ω*_s_ interact with a sample *via* a wave mixing process. Here, the probe beam is commonly at the same frequency as the pump [24]. When the frequency difference between the pump and Stokes beam matches the frequency of a vibrational transition of a molecule, a resonantly enhanced anti-Stokes signal is generated at a frequency *ω*_as_ = 2*ω*_p_ − *ω*_s_ [24]. CARS is typically employed for video-rate imaging of single Raman bands, with most studies focusing on the CH–/OH–stretch region of the spectra for tissue analysis (2500–3500 cm^−1^). Narrow laser bandwidth, speed of laser tuning rates, and nonresonant background interference limit this technique to species with high oscillator density and uniquely isolated Raman peaks [25]. This prevents access to Raman biomarkers in the fingerprint region (500–1800 cm^−1^) of the spectra [25]. Multiplex techniques have been developed to simultaneously excite multiple Raman transitions providing a more complete vibrational picture than found with the single frequency method. With broadband CARS, the pump pulse has a narrow bandwidth and defines the spectral resolution, whereas the Stokes pulse is spectrally broad (usually in the femtosecond regime). Multiple Raman transitions within the bandwidth of the Stokes pulse are excited and are probed. This method allows the entire spectrum of excited states to be obtained at once and has been extended in the fingerprint region of the spectra to allow imaging of biological tissue.
Advantage over standard Raman	Background fluorescence does not interfere with the sample and the signal is 4 orders of magnitude stronger than standard Raman [24].
Disadvantage for intraoperative use	Requires tunable pulsed lasers to probe different molecules in the sample. Difficult to effectively couple and synchronize the lasers into a handheld or portable intraoperative device.
Stimulated Raman scattering (SRS)	
Method	Stimulated Raman scattering typically uses 2 ps pulsed lasers (a pump beam at frequency *ω*_p_ and a Stokes beam at frequency *ω*_s_) that coincide on the sample. By tuning the frequency difference between the pump and Stokes beams to match the frequency of a molecular vibration, *ω*_vib_ = *ω*_p_ − *ω*_s_, stimulated excitation of the vibrational transition occurs [26]. This nonlinear process causes an intensity loss in the pump beam and an intensity gain in the Stokes beam [26]. By modulating one of the beams, e.g., the Stokes beam, and measuring the signal of the pump beam at the frequency of modulation, the intensity loss of the pump beam due to excitation of molecular vibrations can be distinguished from noise to generate high-speed images of a selected Raman band (vibrational transition). While both CARS and SRS occur simultaneously, CARS detects radiation at a new optical frequency, while SRS measures the intensity gain/loss signal of the excitation beams [26, 27].
Advantage over standard Raman	Greater signal strength of approximately 4 orders of magnitude.
Disadvantage for intraoperative use	Coherent techniques such as CARS and SRS allow much more rapid image acquisition than afforded by spontaneous Raman imaging techniques. However, CARS and SERS systems are larger and more complex setups that are difficult to transition to an intraoperative environment. They require tunable pulsed lasers to probe different molecules in the sample, and it may be difficult to effectively couple and synchronize the lasers into a handheld or portable intraoperative device.

## Raman spectroscopy for clinical application

### Neurosurgery

In 2017, there was an estimate of over 23,000 cases of brain and other nervous system cancers in the USA with a 70% mortality rate [28]. According to the most recent data (2010–2014) from the Central Brain Tumor Registry of the United States (CBTRUS), brain and central nervous system cancers were the fifth most common cause of death for ages 15–39 [29]. Glioblastomas, grade IV according to the World Health Organization (WHO), accounted for 14.9% of brain and CNS tumors and 47.1% of malignant tumors with a 4-year survival rate of 7.1% [29]. Petrecca et al. analyzed 20 patients and found that in 17 patients the tumor recurred only at the resection margin; thus, complete tumor resection is crucial for patient longevity [30]. Stummer et al. found that survival for patients with no residual tumor was, on average, 23.6 months; for patients with residual tumors < 1.5 cm survival was, on average, 16.9 months; and patients with residual tumors > 1.5 cm survival was, on average, 13.9 months [31]. This finding underlies the importance of maximum tumor resection during surgery. One of the characteristics of glioblastomas is that it grows in a diffuse manner beyond the primary tumor location. Current image modalities used in presurgical imaging, MRI, do not capture the diffuse nature of glioblastomas. MRI imaging can suffer from brain shift between presurgical pictures and intrasurgery due to gravity, intrasurgical deformation, tumor resection, brain swelling, and cerebrospinal fluid [32, 33]. Raman spectroscopy is a potential modality that can identify the margins of the tumor intraoperatively.

The majority of research into using RS for brain tumor assessment has been done using standard RS [33–45]. Kast et al. and Kalkanis et al. [35, 40] demonstrated RS’s ability to distinguish between white matter, gray matter, glioblastoma, and necrosis. Kast et al. created images from frozen sections of brain tissue samples using Raman peak intensities at 1004, 1300:1344, and 1660 cm^−1^ which are indicative of protein and lipid content. Raman spectra were acquired on five frozen section tissues (one normal, one necrotic, one GBM, and two infiltrating glioma) with an inVia Raman microscope (Renishaw) using an excitation wavelength of 785 nm. The sections were mapped in their entirety using a 300-μm^2^ step size. Smaller regions of interest were also mapped using a 25-μm step size, with each step corresponding to a discrete Raman spectrum. For each Raman image, the pixels were comprised of data from the selected Raman features. Each peak (or peak ratio) was assigned a color: red (1004 cm^−1^), green (1300:1344 cm^−1^), or blue (1660 cm^−1^). The colored images allow interpretation of boundaries between gray matter, white matter, and diseased tissue that corresponded with the findings from adjacent hematoxylin and eosin-stained sections. Performing leave-one-out discriminant function analysis using the three Raman features provided more than 90% classification accuracy [35]. Kalkanis et al. used discriminant functional analysis to distinguish normal tissue, necrosis, and glioblastoma. The Raman spectra from 95 regions from 40 frozen tissue sections were acquired with an inVia Raman microscope (Renishaw) using an excitation wavelength of 785 nm. The spectra were split into a test set, a validation set, and a secondary validation set of tissue with regions containing freeze artifacts. Discriminant function analysis showed 99.6, 97.8, and 77.5% accuracy in distinguishing tissue types in the training, validation, and validation with freeze artifacts datasets, respectively. Decreased classification in the freeze artifacts group was due to tissue preparation damage [40]. Jermyn et al. demonstrated that a handheld RS probe could detect cancer cells intraoperatively that could not be detected by T1-contrast-enhanced and T2-weighted MRI [34]. The gliomas were detected with 93% sensitivity and specificity of 91% [34]. The handheld fiber optic probe (EmVision LLC, FL, USA) was connected to a 785-nm laser (Innovative Photonic Solutions, NH, USA) and a high-resolution charge-coupled device spectroscopic detector (ANDOR Technology, Belfast, UK). The probe was placed in direct contact with the brain at the resection cavity margin for each measurement, with a 0.2-s acquisition time. A supervised machine learning boosted-trees classification algorithm that utilizes all spectral data was used to distinguish samples containing invasive cancer cells *versus* normal brain. The use of a handheld RS probe that can be used intraoperatively is a significant advance and has been used in several studies to successfully identify cancerous cells [33, 34, 37].

Recently, Desroches et al. used a RS needle biopsy system to ensure cells are collected from an area that is dense enough with cancer cells to provide accurate biopsy information, with proof of concept demonstrated during surgery on a pig [45]. Following pig surgery, a different system was used intraoperatively during human glioma surgery to verify that it could detect cancer tissue in biopsy locations [45]. A 671-nm spectrum stabilized near-infrared laser (Laser Quantum, Inc) was used for Raman excitation with spectra collected at 0.5 s acquisition time. Using high wavenumber Raman spectroscopy, dense cancer with > 60% cancer cells was detected *in situ* during surgery with a sensitivity and specificity of 80 and 90%, respectively. The support vector machine (SVM) technique was used for RS tissue classification using 141 features of the spectra. Leave-one-out cross-validation was used to determine the classification accuracy, sensitivity, and specificity. These studies suggest that RS can be used prior to surgery to ensure the biopsy is taken from the correct area and intraoperatively to detect cancerous cells more effectively than current modalities.

Another type of RS being investigated for use during brain surgery is surface-enhanced Raman scattering (SERS) [46–50]. Much of this research is still being completed in animal models due to the requirement of nanoparticles to enhance the surface for RS. Of note, Kircher et al. used a trimodality of MRI, photoacoustic imaging, and SERS in mice to get whole-brain tumor location prior to surgery and during surgery [47]. They measured the Raman signal with a customized Raman microscope (inVia, Renishaw) using an excitation wavelength of 785 nm. Magnetic resonance imaging–photoacoustic imaging–Raman imaging nanoparticles (MPRs) were injected intravenously into glioblastoma-bearing mice. The MPR is a gold-silica–based SERS nanoparticle coated with Gd^3+^ ions. The MPRs accumulated and were retained by the tumors, with no MPR accumulation in the surrounding healthy tissue. The MPRs were detected by all three modalities with at least picomolar sensitivity both *in vitro* and in living mice. Prior to surgery, nanoparticles were visible through the skin and skull of mice to a depth of about 2–5 mm [47]. SERS was used during tumor resection [47]. Residual blood-borne Gd^3+^ was removed by renal function.

Additionally, Karabeber et al. used a handheld Raman probe to detect gold-silica SERS nanoparticles in glioblastoma tumors grown in mice [50]. The particles were intravenously injected into the mice and allowed to circulate for 24 h to ensure that they accumulated in the tumors. Mouse brains were then harvested and fixed in 4% paraformaldehyde. Tumors were then resected with and without Raman guidance. Image guidance with a MiniRam Raman handheld scanner (B&W TEK, Inc., Newark, DE) using a 785-nm excitation laser and 1–2-s long acquisition times was cross-validated with a conventional Raman microscope. The conventional static system was a customized benchtop inVia Raman microscope (Renishaw) equipped with a 785-nm laser as the excitation source with an integration time of 2 s. Both handheld and static SERS image-guided resections were more accurate than resection using white light visualization alone. Correlation with histology showed that SERS nanoparticles accurately outlined the extent of the tumor. Although the Raman scanner cannot acquire the entire SERS images, as with the static system (which takes minutes to hours to map a sample), it has important advantages in that the form factor is conducive for operating room use, it provides near real-time scanning, and it can probe areas of the operative bed due to variable tile angles. The authors demonstrated the handheld probe was able to detect microscopic foci of cancer in the resection bed that were not seen on static SERS images [50]. Although SERS is not as mature as standard RS, it still has considerable potential to be used to detect tumor margins.

Surface-enhanced resonant Raman spectroscopy (SERRS) is another variety of Raman being used to image brain tumors [51–53]. Much like SERS, the research is currently being conducted in animal models, as it requires the use of nanoparticles. Of note, Huang et al. found that the SERRS signal was orders of magnitude higher than nonresonant SERS and is capable of imaging just a few cells [52]. In this study, GBM-bearing mice were intravenously injected with integrin-targeted RGD SERRS nanoparticles. Raman imaging of paraffin-embedded coronal brain sections was accomplished with an inVia Raman microscope (Renishaw) using an excitation wavelength of 785 nm. Integrin targeting was shown to be highly specific to tumor but not normal tissue and enabled visualization of the extent of tumor and the diffuse margin of the main tumor. This also included areas distinct from the main tumor, tracks of migrating cells of two to three cells in diameter and isolated distant tumor cell clusters of less than five cells [52].

Coherent anti-Stokes Raman spectroscopy (CARS) is an alternative type of Raman being investigated to make images of brain tumors. Most studies are being conducted in murine models, but these have recently been extended to human tissue [54–60]. Galli et al. conducted CARS on excised human tissue samples after 5-aminolaevulinic acid (5-ALA) was preoperatively administered. The investigators found that 5-ALA did not interfere with CARS [57]. The fluorescence of 5-ALA-induced protoporphyrin IX was used to identify tumorous tissue. Using it as a reference, CARS images were generated with the signal at a wavenumber of 2850 cm^−1^, which is used to address the distribution of lipids inside tissue. By combining CARS with two-photon excited fluorescence (TPEF) and second harmonic generation (SHG), detailed images of tissue with structures such as extracellular matrix, blood vessels, and cell bodies were produced. The cell morphology in the CARS images was useful for tumor recognition, and the chemical contrast provided by CARS allowed localization of infiltrating tumor cells in fresh tissue samples [57]. Romeike et al. also combined CARS at wavenumber 2850 cm^−1^ with TPEF to produce detailed images of human brain biopsy specimens that had been cryogenically frozen [58]. The images demonstrate cytological and architectural features that may allow tumor typing and grading [58]. They noted that for CARS to advance, it requires miniaturization.

Finally, stimulated Raman spectroscopy (SRS) is a further category of Raman being researched to identify brain tumors [61–65]. Ji et al. used biopsies from adult and pediatric patients to detect tumor infiltration with 97.5% sensitivity and 98.5% specificity with a generalized additive model (GAM) for the classifier [62]. In this method, a Stokes beam (1064 nm) was combined with a tunable pump beam (650–1000 nm) from an optical parametric oscillator that was focused on the sample *via* a laser scanning microscope. The energy difference between the pump and Stokes beams was tuned to specific molecular vibrations, which cause an intensity loss in the pump beam, that are detectable with the aid of a lock-in amplifier. Raman frequencies of 2845 (lipids) cm^−1^ and 2930 (protein) cm^−1^ were chosen for two-color (green, blue) SRS imaging for each 300 × 300 μm^2^ field of view (FOV). Using quantitative measurements of tissue cellularity, axonal density, and protein/lipid ratio in SRS images, they derived a classifier capable of detecting tumor infiltration [62]. Hollon et al. also used fresh tissue from pediatric patients with classification algorithm accuracy of 93.8% on cross-validated data on normal *versus* lesional tissue and 89.4% accuracy on cross-validated data for low-grade *versus* high-grade tumors [63]. SRS images were generated with a clinical fiber-laser–based SRS microscope. Raman frequencies of 2845 (lipids) cm^−1^ and 2930 (protein) cm^−1^ were chosen for two-color (green, blue) 400 × 400-μm^2^ SRS images. These images allow neuropathologists to diagnose the tissue with 92–96% accuracy. The image features were then used to develop a random forest machine learning model for automated classification [63]. Lu et al. profiled 41 specimens resected from 12 patients with a range of brain tumors. SRS Raman imaging data were correlated with the current clinical gold standard of histopathology and were shown to capture many essential diagnostic hallmarks for glioma classification. Interestingly, in fresh tumor samples, Lu et al. detected structures that were not evident in the H&E stains, such as abundant intracellular lipid droplets within the glioma cells, collagen deposition in gliosarcoma, and irregularity in the disruption of myelinated fibers in areas infiltrated by oligodendroglioma cells [64].

Lastly, progress is being made in making SRS more portable and practical for the surgical suite. Orringer et al. demonstrated SRS microscopy in the operating room using a portable fiber-laser–based microscope and unprocessed specimens from 101 neurosurgical patients [65]. Histologic images of fresh, unstained surgical specimens were created with the clinical SRS microscope. The all fiber-based system had a 790-nm pump beam and a tunable Stokes beam over the entire tuning range from 1010 to 1040 nm. While for clinical implementation an all fiber system is desired, the relative intensity of noise intrinsic to fiber lasers can vastly degrade SRS image quality. To address this, the authors developed a noise cancelation scheme to improve the signal-to-noise ratio by 25-fold. Images were created by mapping two biologically significant Raman shifts: 2845 cm^−1^, which corresponds to CH_2_ bonds in lipids, and 2930 cm^−1^, which corresponds to CH_3_ bonds in proteins and DNA. To produce simulated Raman histology (SRH) images, FOVs are acquired at a speed of 2 s per frame in a mosaic pattern, stitched, and recolored. A subtracted CH_3_–CH_2_ image was assigned to a blue channel and a CH_2_ image was assigned to the green channel. Using SRH images generated by this system, pathologists diagnosed lesional from nonlesional areas with 98% accuracy and glial from nonglial tumors with 100% accuracy [65]. The authors employed a machine learning process called a multilayer perceptron (MLP) for diagnostic prediction. The diagnostic capacity for classifying individual FOVs as lesional or nonlesional was 94.1% specificity and 94.5% sensitivity, and glial from nonglial specimens were differentiated with 90% accuracy [65]. With this advance, SRS is now a promising technology for identifying tumor margins in brain cancer. Neuronavigation techniques and brain tumor assessment can benefit from the addition of Raman spectroscopy systems during surgery.

### Ovarian cancer

Ovarian cancer is the fifth leading cause of cancer among women. In 2018, the estimated number of women that will receive a new diagnosis of ovarian cancer in the USA is about 22,240 with 14,070 estimated deaths [66]. Advances in identifying precursor lesions to ovarian cancer may enhance the ability to detect early-stage disease. The 5-year relative survival rate for women treated for cancer at stages IA and IB is 92%. However, only 15% of all ovarian cancers are found at this early stage [67]. Scientists at the ovarian cancer SPORE at Brigham and Women’s Hospital have found evidence that a majority of serous cancers originate in the fimbria of the fallopian tube rather than on the ovarian surface [68].

#### Raman spectroscopy of ovarian cancer tissues

The authors did a preliminary investigation on excised fallopian tubes to identify spectral biomarkers that distinguish cancer from normal tissue. Fresh tissue samples from surgical resection were used to generate immunohistochemical profiles and Raman spectra of the inner wall of fallopian tubes from normal tissue and from malignant carcinomas. The Raman spectra were acquired with an inVia Raman microscope (Renishaw) using an excitation wavelength of 785 nm. Several spectral biomarkers (indicative of protein and lipid changes) were relevant in distinguishing between healthy fallopian tube, malignant ovary, and omental metastasis. The Raman bands were located at wavenumbers of 718, 1004, 1090, 1247, 1321, 1340, 1440, and 1660 cm^−1^. These correspond to: (a) C–N vibrations of membrane phospholipid head; (b) symmetric ring breathing mode of phenylalanine; (c) symmetric phosphate stretching vibrations of DNA/C–N stretching of protein; (d) amide III vibrations; (e) CH_3_CH_2_ twisting in collagen/amide III vibrations; (f) nucleic acids/collagen; (g) CH_2_, CH_3_ deformations of lipids and collagen; and (h) amide I vibrations, respectively. These spectroscopic biomarkers may provide insight on the evolution of the disease.

Another study used fresh ovarian tissue samples from biopsy or surgical resection in saline solution from the Department of Obstetrics and Gynecology, Manipal University, Manipal [69]. In this study, a 785-nm diode laser was used for excitation and the Raman signals were detected by an HR 320 spectrograph. A holographic filter and a notch filter were used to filter out unwanted lines from the excitation source and reject Rayleigh scattering from the Raman signals, respectively. The scientists obtained 72 certified spectra, 38 spectra of eight normal tissues, and 34 spectra of seven malignant tissues. Grams 32 software was used to carry out baseline correction, smoothening, calibration, and normalization over *δ*CH_2_. Grams PLS Plus/IQ was used to carry out principal components analysis (PCA) in the 800–1800-cm^−1^ spectral range. Following analysis, the spectral features of the malignant tissues revealed the presence of additional biomarkers including proteins, lipids, and DNA. These were defined by a broader amine I band (protein), stronger amide III band (protein), a minor blue shift in the *δ*CH_2_ band (lipid), and a hump around 1480 cm^−1^ (DNA) and other peaks around 834, 900, 1000, and 1165 cm^−1^ (proteins) when compared to normal tissue spectra [69]. The study also found that variations in the secondary structures of proteins were implicated by spectral profiles in the 900–950-cm^−1^ region [70, 71]. Multiple methods were employed for analysis including discriminating algorithms, score of factor, Mahalanobis distance, spectral residuals, and the limit test. Maheedhar et al. were able to obtain a 100% specificity and sensitivity using the limit test approach. Moreover, the results provided unambiguous and objective discrimination. The method is easily adaptable to routine clinical conditions and facilitates diagnosis of ovarian cancers by minimally skilled personnel making it more cost-effective.

Raman spectroscopy has been utilized to detect metastasis [72]. The primary metastatic route of ovarian cancer occurs through the peritoneal surface [73]. The metastasis begins at the microscopic level and, therefore, can be easily missed during investigation of possible tumors. In addition, the folate receptor (FR) is overexpressed in more than 70% of primary ovarian cancers [74]. Researchers at the Department of Radiology and Center for Molecular Imaging and Nanotechnology developed a folate-targeted detection method of microscopic ovarian tumors [72]. The method utilizes SERRS nanoparticles (NP) to enhance the weak Raman signals, and increase sensitivity and specificity for detection of the folate receptors, which are indicative of ovarian cancer metastasis. Two types of NPs were synthesized with gold nanostar cores and silica shells: a targeted nanoprobe functionalized with an anti-folate receptor antibody (αFR-Ab) *via* a PEG–maleimide–succinimide crosslinker and using the infrared dye IR780 as the Raman reporter and a nano-targeted probe (nt-NP) coated with PEG_5000_-maleimide and containing IR140 infrared dye as the Raman reporter. Mouse studies were conducted on athymic mice. The mice were injected with 4 × 10^6^ SKOV-3 cells transduced with luciferase and green fluorescent protein. The NPs were injected intraperitoneally, which prevented systemic uptake. Luciferin was then injected retro-orbitally. The entire mouse was imaged with BLI and the regions of interest were scanned with the Raman microscope to assess the correlation of the ratiometric method to the BLI map. The ratiometric method was shown to be accurate for screening metastasis at the microscopic level and tumors as small as 370 μm were found [72]. This method was called “topically applied surface-enhanced resonance Raman ratiometric spectroscopy”, or TAS3RS for short.

#### Raman spectroscopy of ovarian cancer patient serum

Screening to detect early-stage ovarian cancer is difficult due to the lack of symptoms or minimal nonspecific symptoms early in the disease. Until now, no tumor markers have been identified with the high sensitivity and high specificity necessary to develop a screening test for ovarian cancer. Serum markers, such as cancer antigen (CA-125), are often used in clinical practice. CA-125 is often elevated in women with advanced ovarian cancer. However, this marker is tumor-associated rather than tumor-specific and lacks the specificity and sensitivity required for early detection. The FDA-approved OVA1 measures five biomarkers in the blood to assess the likelihood of ovarian cancer in women diagnosed with ovarian mass that requires surgery. OVA1 has been shown to have over 90% sensitivity but low specificity (~ 35%) with a positive predictive value of 40% [75]. ROMA (The Risk of Ovarian Malignancy Algorithm) evaluates HE4 (a whey acidic four-disulfide core domain protein) and CA-125 levels along with a woman’s menopausal states to generate scores. The scores reflect a predictive index of ovarian cancer for women diagnosed with ovarian tumor that requires surgery. ROMA has reported 89% sensitivity and has a specificity of 75% [75].

CA-125 is elevated by 23–50% in stage I and 90% in stage II ovarian cancer patients. CA-125 detection has poor sensitivity and specificity for ovarian cancer making it a poor screening target when used alone [76]. However, the detection of other biomarkers paired with detection of CA-125 could be much more effective. Researchers at the Pakistan Institute of Engineering and Applied Science, National Institute of Lasers and Optronics, and Citi Lab conducted a study investigating optical differences between the serum of healthy and ovarian cancer patients using Raman spectroscopy [77]. In this study, blood samples from 11 patients with confirmed clinical and histopathological ovarian cancer and 11 healthy volunteers that matched the case group in demographic profile including median age, race, and gender were used to study the possible spectroscopic signatures of ovarian cancer compared to healthy samples. The sera were extracted and stored at − 20 °C until final Raman spectroscopic measurement. The researchers obtained 42 Raman spectra using Raman spectrometer (Dongwoo Optron). They used a 532-nm wavelength light beam for probing the samples. A ×100 objective lens was used to properly direct the incident light on the sample and to focus the light after interaction on the detector in the backscattering configuration. Raman spectra were obtained for each sample in the spectral range of 500–2000 cm^−1^. A Savitzky–Golay (SG) was used to improve the signal-to-noise ratio (SNR) while preserving the integrity of the weak Raman peaks. The cubic spine interpolation method followed by spectra normalization was used to remove the fluorescence contribution toward the Raman spectra. The researchers developed a SVM algorithm toward computer-assisted classification of healthy and ovarian cancer samples based on the differences in Raman spectra. The algorithm first calculates the *p* values from unpaired two-tailed *t* tests and categorizes the spectra into three categories: *p* < 0.05 (five peaks), *p* < 0.01 (one peak), and *p* < 0.0001 (six peaks). Four samples from each group were then used to train the SVM algorithm for blind classification of the remaining samples. Lastly, the performance of the algorithm was evaluated in terms of specificity, sensitivity, positive predictive values, and negative predictive values. An unpaired samples *t* test was used to analyze differences in amplitude and peak positions and showed significant results. The maximum amplitude differences on the spectra were the CH_2_ peak at 1447 cm^−1^, the amide peak at 1657 cm^−1^, and the C=O stretching peak at 1744 cm^−1^, which were assigned to the bending of proteins/lipids/fatty acids, the amide I stretching of protein backbone, and the stretching of lipids, respectively [77–79]. Amplitude peaks were also found at 640, 749, and 950 cm^−1^, which were allotted to the stretching of C–S in cysteine, the symmetric breathing of tryptophan, and the hydroxyapatite/carotenoid/cholesterol breathing of phenylalanine, respectively [77, 78]. The observed differences in peak amplitudes and positions found in this study can be attributed to changes in expression of multiple proteins as well as changes to protein conformation. In ovarian cancer, CA-125, human epididymis protein 4 (HE4), haptoglobin, osteopontin, and mesothelin, among other proteins are overexpressed [80–83]. As previously stated, the peak position differences were divided into three categories, with 846 cm^−1^ as the only member of *p* < 0.01 group. The evaluation of the SVM algorithm showed encouraging results with a sensitivity of 90%, specificity of 100%, positive predictive values of 100%, and negative predicted value of 87.5%, when the combination of all spectral peaks (*p* < 0.05, *p* < 0.01, *p* < 0.0001) was used.

#### Raman spectroscopy of ovarian cancer cell lines

Raman spectroscopy can not only help with the discrimination between malignant and healthy tissue and between malignant and healthy serum, but it has also been used to discriminate between chemically fixed cisplatin-resistant (A2780cp) and cisplatin-sensitive (A2780s) human ovarian carcinoma cells. Most patients initially respond to chemotherapy; however, about 75% of those patients relapse after treatment, and about 30% will fail to respond to treatment and/or quickly progress over the course of 1 year of treatment [84]. The main reason behind the relapses is that prior to treatment, some cancer cells were platinum-resistant. Following treatment, the platinum-sensitive cells are destroyed while the platinum-resistant cells continue to multiply, thus creating a platinum-resistant tumor. The ability to remove the platinum-resistant tumor in the early stages would improve prognosis. Researchers at Carleton University used Raman spectroscopy to differentiate between cultured A2780s and A2780cp cells. They fixed the cells to coverslips in order to preserve the cells prior to and during Raman imaging. Imaging was performed with a confocal Raman microscope with a 785-nm diode laser. The light was reflected off a dichroic mirror, and the reflected light passed through a ×60 water-immersion objective with a numerical aperture (NA) of 1.5 and focused to a spot of diameter ~ 1 μm. The backscattered light was filtered to remove the Rayleigh-scattered laser light and focused into a 100-μm pinhole. The light collected from the focal plane was directed to a Shamrock 303i-B spectrograph. This provided a spectral range from 700 to 1600 cm^−1^. The Raman spectra were collected using a CCD camera that was thermoelectrically cooled to − 80 °C. The spectra underwent background subtraction, normalization, and noise reduction to obtain accurate Raman peaks of the cells for subsequent cells. Background subtraction was carried out using a modified version of the open source algorithm (SMIRF) from the University of Rochester [85]. A Savitzky–Golay filter was used to smooth the spectra. PCA, combined with linear discriminant analysis (LDA), was performed on the Raman spectra for classification purposes. The relative abundance of proteins and glutathione in the A2780cp compared to the A2780s cells is a strong indicator of platinum resistance. The main peak contributions to this discrimination were at 746, 849, 873, 1002, 1030, 1176, 1208, 1553, and 1584 cm^−1^, which were all spectral features of proteins arising from aromatic amino acids such as tyrosine, phenylalanine, and tryptophan. Peaks at 932, 955, 983, 1086, and 1158 cm^−1^ were due to carbon stretching or deformation of carbon atoms bonded with other nitrogen or carbon atoms. The peaks at 932 and 1441 cm^−1^ can also be attributed to the vibration of glutathione, which has also been associated with resistance to cisplatin in A2780cp cells [86–89]. The spectra contained peaks at 782, 810, 1338, and 1579 cm^−1^, which were due to vibrations of individual DNA/RNA bases. Vibrations due to lipids were shown corresponding to peaks 718, 824, 1064, and 1302 cm^−1^ with some overlap at 1127 cm^−1^. The spectra of the A2780s and A2780cp cells were mostly identical with a few notable differences at 718, 932, 1086, 1127, 1262, 1301, and 1335 cm^−1^, which are attributed to protein, nucleic acid, and lipid spectral features mentioned previously.

### Prostate cancer

Prostate cancer is the second leading cause of cancer-related death in the USA. About one in nine men will be diagnosed with prostate cancer in their lifetime with the average age at diagnosis being about 66 [90]. It is estimated that in 2018 about 164,690 men will be diagnosed with prostate cancer with 29,430 estimated deaths. When treated, the 5-, 10-, and 15-year survival rate is 99, 98, and 96%, respectively [91]. In 2014, Kast et al. discussed the clinical applications of Raman spectroscopy to prostate cancer including screening, biopsy, margin assessment, and monitoring of treatment efficacy as well as potential future avenues of research with emphasis on multiplexing Raman spectroscopy with other modalities [92]. Along with similar reviews [93, 94], they found successful clinical proof-of-concept, surgical RS fiber optic probe studies for a variety of other cancers *in vivo*, including bladder, breast, colon and upper GI, lung, brain, skin, and cervical. However, up to that point, only *in vitro* studies on surgical RS fiber optic probes for prostate cancer had been conducted.

#### Raman spectroscopy of prostate cancer cell lines

Since Kast et al., progress has been made in using various types of RS in clinical settings for the detection and diagnosis of prostate cancer. Beginning with *ex vivo* pathology, Corsetti et al. took advantage of RS’s high chemical specificity to differentiate between a late-stage androgen-resistant cancer cell line from a nonandrogen-resistant line. A custom-made Raman setup using a 785-nm fiber-coupled diode laser was narrowed to an 18-μm output so that a single acquisition was representative of a single cell. Three spectral regions were acquired in succession for each cell: the “fingerprint region” (330–1350 cm^−1^), the “bending region” (1400–1800 cm^−1^), and the “stretching region” (2800–3100 cm^−1^). Spectral data were analyzed using PCA and subsequent LDA to the fingerprint region resulting in cell line differentiation with 95% sensitivity and 88% specificity *via* phenylalanine, tyrosine, DNA, amide III, and l-arginine content [95]. Aubertin et al. differentiated between benign and malignant prostate tissue biopsies of 32 patients using a custom handheld contact RS probe system. The probe consisted of seven 300 μm core detection fibers surrounding a 272-μm core excitation fiber through which a wavelength-stabilized 785-nm laser light was passed. Spectral data were classified using supervised machine learning neural network methods with leave-one-out cross-validation. The entire spectrum (500–1700 cm^−1^) was used to distinguish benign and malignant tissue samples, among other histopathological criterions, with a sensitivity of 87% and a specificity of 86% [96]. In addition, Lernhardt et al. presented that they had success in distinguishing between aggressive and nonaggressive prostatectomy cancer tissue in a retrospective study of 30 prostatectomy patients of known outcome using a Raman confocal microspectrometer (CellTool BioRamTM) with an accuracy of 84% [97].

#### Raman spectroscopy of prostate cancer patient blood and plasma samples

Moving away from invasive biopsy-based methods, Li et al. demonstrated potential for a noninvasive prostate cancer screening technology using silver colloidal SERS nanoparticles mixed with serum from peripheral blood samples of 68 healthy volunteers and 93 histology-confirmed prostate cancer patients. Spectra were collected using a Renishaw Raman system (inVia) with a 785-nm diode laser and normalized in the range of 400–1800 cm^−1^. Conventional SVM diagnostic algorithms were developed to classify serum SERS spectra between cancer and normal with a 98.1% diagnostic accuracy [98]. Later, Medipally et al. tested a high-throughput RS technique on peripheral blood plasma using a Horiba (Jobin Yvon LabRAM HR 800) setup equipped with 785, 660, 532, and 473 nm lasers collecting spectra from each sample in a range of 400–1800 cm^−1^. After preprocessing, spectra were analyzed using PCA–LDA with leave-one-out cross-validation that differentiated between prostate cancer patients and noncancer controls with a sensitivity and specificity of 96.5 and 95%, respectively [99]. Furthermore, a preliminary study by Del Mistro et al. has shown the potential for gold nanoparticle-based SERS as a noninvasive prostate cancer screening technology *via* urine sample interrogated with a 785-nm laser through a Renishaw Raman (inVia) setup. Preprocessing was conducted using hyperSpec package for R and classified using a PCA–LDA model with a sensitivity of 100%, a specificity of 89%, and an overall diagnostic accuracy of 95% [100].

#### Raman spectroscopy of prostate tissues

To more closely address surgical needs, Harmsen et al. demonstrated successful proof-of-concept prostate cancer margin demarcation in near-clinical situations on a mouse model using a SERRS nanostars and RS imaging combination. Spectra were collected using a Renishaw Raman (inVia) system equipped with a 785-nm diode laser, and statistical significance was determined with a Student’s *t* test [101]. While nanoparticle injection methods have many obstacles including cytotoxicity and FDA clearance [102], Harmsen et al. showed that tumor boundaries could be detected in near real time under clinically viable 10–100 mW laser power. Alternatively, Lindahl et al. have proposed a dual RS and stiffness sensing probe intended for detecting any positive surgical margin left behind during radical prostatectomy. The probe consists of a hollow stiffness sensor through which fiber optics are fed and connected to a 785-nm RXN1 Raman spectroscope (Kaiser Optical), allowing the user to switch between modalities without moving the probe. A total of 36 measurements were taken *ex vivo* on four radical prostatectomy human prostates. Stiffness, autofluorescence, and the Raman peak found at 2881 cm^−1^ were used as discriminatory parameters. However, strong Raman fluorescence resulted in lower detectability (77% sensitivity and 65% specificity). Yet, with stiffness and autofluorescence parameters combined, they were able to achieve 100% sensitivity and 91% specificity, demonstrating potential utility of the probe’s combination [103, 104]. Using a non-SERS probe would eliminate the need for nanoparticle injection if they can overcome the tissue’s inherently strong fluorescent background.

Overall, various RS technologies continue to demonstrate great promise as a noninvasive prostate cancer diagnostic tool. However, there is much room for technique and optics improvement before being used in the surgical suite, specifically optimizing excitation wavelength to mitigate inherent fluorescence of prostate tissue.

### Pancreatic cancer

Pancreatic cancer is the third leading cause of cancer-related death in the USA [105]. It is estimated that in 2018 about 53,600 people will be diagnosed with pancreatic cancer with 44,300 estimated deaths. The 5-year survival rate of people treated for exocrine pancreatic cancer at stage IA is 12%, stage IIA 5%, stage III 3%, and stage IV 1% [106]. The survival rate of patients with neuroendocrine pancreatic tumors that were treated with surgery at stage I is 61%, stage II 52%, stage III 41%, and stage IV 16%. About 94% of pancreatic cancers are classified as exocrine tumors with the vast majority being adenocarcinomas [107].

Scientists at the Leibniz Institute of Photonic Technology and Friedrich Schiller University Jena collected Raman spectra from T lymphocyte Jurkat cells and pancreatic cell lines Capan1 and MiaPaca2 [108]. Their Raman microscopy setup uses a 785-nm single-mode excitation laser and a sample holder mounted to a motorized x–y translational stage with a manual Z-positioning stage. An oil immersion objective lens focuses the excitation laser beam into the sample plane to a spot size of approximately 0.8 μm with a focal length near 1.6 μm. The spectrometer resolution is 9 cm^−1^ from 300 to 4000 cm^−1^ range. The Raman signal is received by a CCD with 400 × 1340 pixels. Using a support vector machine method with linear kernel, coupled with PCA, the cell classification precision for pancreatic cell lines is higher than 90%. They found that pancreatic cells have higher lipid content, which is evident from stronger lipid-related bands in the high wavenumber region at 2854 cm^−1^, and higher band ratios 1440/1660 and 1320/1340 cm^−1^. Also, the acquisition of integrated Raman signals of large portions of cells allowed for sampling of single cells and simpler interpretation of the cell type differences that are comparable to the acquisition of single spectra. The integrated Raman spectra approach provided better and more stable predictions for individual cells and may have a major impact on the implementation of Raman-based cell classification.

Researchers from Purdue University and Indiana University School of Medicine found a link between cholesterol esterification and metastasis in pancreatic cancer. They used SRS microscopy and Raman spectroscopy to map lipid droplets (LDs) stored inside single cells. Analyses of the composition of individual LDs revealed an aberrant accumulation of cholesteryl ester (CE) in human pancreatic cancer specimens and cell lines [109]. Their SRS imaging was conducted using a femtosecond laser source. The pump and Stokes beams are collinearly overlapped and combined with the pump beam that is tunable from 680 to 1080 nm and the Stokes beam that is tunable from 1.0 to 1.6 μm. Images were taken on a laser scanning microscope with a ×60 water immersion objective. The signals were detected by a photodiode and then sent to a fast lock-in amplifier, which has a time constant as small as 800 ns. The lateral and axial resolutions of their SRS microscope are about 0.42 and 1.01 μm, respectively. For coherent Raman scattering imaging, two synchronized 5-ps, 80-MHz laser oscillators are temporally synchronized and collinearly combined into a laser scanning inverted microscope. The CARS signals are detected by photomultiplier tube detectors. Confocal Raman microspectroscopy is realized by mounting a spectrometer to the side port of the microscope. The pump and Stokes lasers are tuned to 707 and 885 nm, respectively, to be in resonance with the CH2 symmetric stretch vibration. The spectrometer is equipped with a 300-grooves/mm 500-nm blaze angle grating and a thermoelectrically (TE) cooled back-illuminated electron-multiplying charge-coupled device. LD amount was quantified based on the SRS images using the software ImageJ. CE level in individual LDs was quantified by analyzing the height ratio of the 702-cm^−1^ peak to 1442-cm^−1^ peak. They found that the peak of cholesterol at 702 cm^−1^ and the peak of ester bond at 1742 cm^−1^ are high for cancer tissues. They also found that abrogation of cholesterol esterification, either by an ACAT-1 inhibitor or by shRNA knockdown, significantly suppressed tumor growth and metastasis in an orthotopic mouse model of pancreatic cancer. These results demonstrate a new strategy for treating metastatic pancreatic cancer by inhibiting cholesterol esterification.

About 10 years prior, researchers at Wayne State University [110] collected Raman spectra of normal and pancreatic tissue from mouse model using a Renishaw Raman microscope equipped with a thermoelectric cooled 578 × 385-pixel CCD. A 785-nm wavelength laser line (approximately measured at 130 × 25 um) was used to excite the tissue sample with 50 mW of power. The excitation laser line covers a section of tissue encompassing multiple cells and reflects the averaged characteristic over that section. The spectral range is from 600 to 1800 cm^−1^, with the resolution of 4 cm^−1^. The Raman data were analyzed by PCA and discriminant function analysis (DFA). They found that Raman spectroscopy differentiated normal pancreatic tissue from tumors in a mouse model with high sensitivity (91%) and specificity (88%), and pancreatic tumors were characterized by increased collagen content and decreased DNA, RNA, and lipid components compared to normal pancreatic tissue.

Using SERRS nanoparticles, scientists at the Memorial Sloan Kettering Cancer Center demonstrated an imaging method for the precise visualization of tumor margins, microscopic tumor invasion, and multifocal locoregional tumor spread [111]. They designed, synthesized, and tested a new SERRS nanoprobe that is resonant in the near-infrared (NIR) window, where optical penetration in tissue is maximized. Their nanoparticles feature a star-shaped gold core, a Raman reporter resonant in the near-infrared spectrum, and a primer-free silication method. Raman scans were performed on an inVia Raman microscope (Renishaw) equipped with 785 nm diode laser and a 1-in. charge-coupled device detector with a spectral resolution of 1.07 cm^−1^. The Raman maps were generated and analyzed by applying a DCLS algorithm (WiRE 3.4 software, Renishaw). Counts per second represent the intensity of the 950-cm^−1^ peak of SERRS nanoparticles. Statistical analysis was performed in Excel (Microsoft). In genetically engineered mouse models of pancreatic cancer, breast cancer, prostate cancer, and sarcoma, and in one human sarcoma xenograft model, this method enabled accurate detection of macroscopic malignant lesions, as well as microscopic disease, without the need for a targeting moiety, and the sensitivity (1.5 fM limit of detection) of this method allowed imaging of premalignant lesions of pancreatic and prostatic neoplasias.

#### Raman spectroscopy of pancreatic cancer serum markers

Early-stage pancreatic cancer is difficult to detect due to the lack of symptoms, which often results in diagnosis at an advanced stage of disease. CA19-9 and carcinoembryonic antigen (CEA) are tumor markers that may be detected in the blood and are tied to pancreatic cancer. These proteins may or may not be elevated in a person with pancreatic cancer. About 59% of patients with pancreatic carcinoma have high concentrations of CEA that suggest a mucinous pancreatic cyst. However, CEA testing does not reliably distinguish between begin, premalignant, or malignant mucinous cysts. Serum CA19-9 is a tumor-associated mucin glycoprotein antigen related to the Lewis blood group protein. About 5% of the population do not produce CA19-9 antigen. The sensitivity (68–93%) and specificity (76–100%) of CA19-9 is not adequate for diagnosis and precludes it as a screening tool [112].

Using SERS, researchers from Iowa State University, University of Nebraska Medical Center, University of Pittsburgh Medical Center, and University of Utah demonstrate the first ever detection of the potential pancreatic cancer marker MUC4 in cancer patient serum samples [113]. Their SERS-based immunoassay chip design includes (a) a capture substrate to specifically extract and concentrate antigens from solution, (b) surface-functionalized gold nanoparticles (extrinsic Raman labels or ERLs) to bind to captured antigens selectively and generate intense SERS signals, and (c) sandwich immunoassay with SERS readout. The Raman spectra were collected with a NanoRaman I fiber-optic–based Raman system, a portable, field-deployable instrument. The light source was 632.8 nm He–Ne laser. The spectrograph consisted of an imaging spectrometer (6–8 cm^−1^ resolution) and a CCD imaging array. The incident laser light was focused to a 25-μm spot on the substrate. The analyte concentration was quantified using the peak intensity of the symmetric nitro stretch at 1336 cm^−1^. The amount of human mucin MUC4 was measured in CD18/HPAF lysate (positive control) by sandwich enzyme-linked immunosorbent assay (ELISA). SERS measurements showed that sera from patients with pancreatic cancer produced a significantly higher SERS response for MUC4 compared to sera from healthy individuals and from patients with benign diseases. And SERS measurement can also detect CA19-9 concentration.

Recently, scientists at the University of Massachusetts [114] demonstrated a novel system for multiplex detection of pancreatic biomarkers CA19-9, MMP7, and MUC4 in serum samples with high sensitivity using surface-enhanced Raman spectroscopy. Their SERS-based immunoassay for biomarker quantification includes (I) functionalizing gold substrate with thiol and antibody, (II) capturing desired antigens from the serum, and (III) loading antibody-conjugated extrinsic Raman labels (ERL), and gold nanoparticles were modified with antibody and Raman reporter. Raman spectra collection was performed with a portable BWS415 i-Raman at an excitation wavelength of 785 nm. The antigen concentration was quantified using intensity at the 1336-cm^−1^ position which corresponds to a symmetric stretch of the NO_2_ group whose intensity of this band depends proportionally on the concentration of MUC4 in a sample. They found that immobilization of functionalized gold nanoshells with resonance wavelength of 660 nm on the gold-coated silicon substrate led to a significant improvement of SERS signals, and successfully detected three pancreatic biomarkers, CA19-9, MMP7, and MUC4, in spiked serum samples at concentrations as low as 2 ng per ml. Measuring the levels of these biomarkers in pancreatic cancer patients, pancreatitis patients, and healthy individuals revealed the unique expression pattern of these markers in pancreatic cancer patients, suggesting the great potential of using this approach for early diagnostics of pancreatic cancers.

### Breast cancer

Breast cancer is the leading cause of new cancer cases (30% of all new cancer cases) and the second leading cause of cancer deaths (14% of all cancer deaths) in American women [115]. A low-dose X-ray mammogram is the most common technique used for screening of microcalcifications in breast cancers. Mammography is not effective in dense female breasts and do not discriminate whether a lesion is benign or malignant. Therefore, it is always followed by either surgical excision biopsy or needle biopsy, thus delaying the diagnostic process from weeks to months causing unnecessary psychological stress and medical costs. The combination of clinical breast examination, mammography, and tissue sampling together is effective in improving the sensitivity and specificity of breast cancer detection [116–118]. However, only 36.5% of microcalcifications identified on a mammogram that are subsequently biopsied turn out to be malignant [119]. There is a need for minimally invasive optical imaging and spectroscopy techniques that can improve breast cancer diagnosis, especially with the ability to distinguish benign from malignant breast tissues.

#### Raman spectroscopy of breast cancer tissues

Alfano et al. is the first group to use FT Raman spectroscopy with 1064 nm laser excitation source on three normal, four benign, and seven malignant breast tissues. They observed spectral variation between three different breast tissues and correlated these to differences in amide modes [120]. Using the same methodology (FT RS with 1064 nm laser excitation), Bitar et al. in 2006 tried to differentiate normal and six subtypes of breast pathologies: fibrocystic condition, ductal carcinoma *in situ*, ductal carcinoma *in situ* with necrosis, infiltrating ductal carcinoma not otherwise specified (NOS), colloid infiltrating ductal carcinoma, and invasive lobular carcinomas. Except for inflammatory and medullary ductal carcinomas from infiltrating duct carcinoma NOS, they were able to differentiate normal tissue from diseased breast tissue subtypes based on spectral differences. By relating each observed peak to a specific biomolecule with a special role in carcinogenesis, they established biochemical basis for each spectrum [121].

Using conventional Raman spectroscopy, Redd et al. in 1993 studied the Raman spectra of normal, benign, and malignant breast tissue using different excitation wavelengths of the visible region (406.7, 457.9, and 514.5 nm). The peak differences between benign and malignant breast tissues were attributed to β-carotene and fatty acids [122]. In 1995, the same group used a 784-nm excitation source to differentiate normal breast tissue from benign (fibroadenoma) and malignant tissues (infiltrating duct carcinoma NOS). Using the area ratio of amide I and CH_2_ bending modes as a discriminating parameter, they established profiles for normal tissue (more lipids, mainly derivates of oleic acids) and malignant tissues (increased protein content). However, their study could not statistically differentiate infiltrating duct carcinoma and fibroadenoma [123]. Manoharan et al. in 1996 used NIR Raman spectroscopy with an 830-nm excitation source to examine normal, fibroadenoma, or infiltrating duct carcinoma NOS breast tissues. They used ratio of amide I to CH_2_ bending modes as their discriminating parameter and employed PCA and multivariate analysis for statistical analysis. They also observed abundance of lipid features in normal breast tissue spectra and abundance of protein signatures in breast lesion spectra. However, they could not differentiate benign from malignant tissues [124].

Haka et al. in 2002 used a confocal micro-Raman spectroscope with an 830-nm excitation source on breast tissues. They used a microlaser spot (~ 5–20 μm instead of ~ 50–100 μm) and constructed a morphological/chemical model for the breast tissue by fitting tissue spectra with a linear combination of basis spectra derived from cell cytoplasm, cell nucleolus, fatty acids, β-carotene, collagen, calcium hydroxyapatite, calcium oxalate dehydrate, cholesterol-like lipids, and water. For their diagnostic algorithm, they used fit coefficients of fat and collagen and reported an abundance of lipids in normal breast tissue, increased levels of collagen in diseased breast tissue, and markedly elevated levels in benign conditions [125]. They adopted this approach to *ex vivo* diagnosis and classified benign and malignant breast legions with 94% sensitivity and 96% specificity [126]. Further, they adopted this approach to *in vivo* intraoperative tumor margin assessment. They collected 31 Raman spectra from nine patients undergoing partial mastectomy procedures and fit the data into their previously established model, which resulted in characterization of tissue in 1 s. Using this method, they were able to detect grossly invisible cancer that was validated later by pathology review [127]. Mohs et al. in 2010 used a different approach in which they used Raman spectroscopy to measure exogenous contrast agents that were designed to adhere to tumor cells during surgical procedures for *in vivo* and intraoperative tumor detection. They developed a handheld spectroscopic device named “SpectroPen” that has a 785-nm laser source and is coupled with compact head unit for light excitation and collection. Using SpectroPen, they detected *in vivo* fluorescent contrast agent (indocyanine green, ICG) with a limit of detection of 2–5 × 10^−11^ M and SERS contrast agent (pegylated colloidal gold) with a limit of detection of 0.5–1 × 10^−13^ M with a tissue penetration depth of 5–10 mm [128].

In another study, Haka et al. investigated the chemical composition of macrocalcifications in breast duct. They showed that microcalcifications can be divided into type I, consisting of calcium oxalate dihydrate that are present in benign lesions, and type II, consisting of calcium hydroxyapatite deposits that are present in proliferative lesions, which can be either benign or malignant depending on their carbonate content. Benign lesions had more calcium carbonate and less proteins compared to malignant lesions. PCA and logistic regression analysis demonstrated 88% sensitivity and 93% specificity in diagnosing type II microcalcifications which is a significant improvement over X-ray mammography [125]. They adopted this technique to detect microcalcifications in core needle biopsies using a portable, compact clinical Raman spectroscopy system, which has 830 nm excitation source. Using this system, they tested 159 tissues samples from 33 patients (54 normal, 75 lesions with microcalcifications, and 30 lesions without microcalcifications) and obtained 97% of positive predictive value in correctly classifying microcalcifications [129]. Further, this portable Raman spectroscope system was used as a guidance tool for mastectomy procedures. From 33 patients that underwent mastectomy, 146 freshly excised tissue sites (50 normal, 77 lesions with microcalcifications, and 19 lesions without microcalcifications) were used *ex vivo* to obtain Raman spectra. They reported 62.5% sensitivity and 100% specificity and showed potential of Raman spectroscopy to provide real-time feedback and simultaneously detect microcalcifications and diagnose associated lesions, including ductal carcinoma *in situ* [130].

Baker et al. and Matousek et al. in 2007 used Raman spectroscopy to noninvasively detect microcalcifications *in vivo*. Using Kerr-gated Raman spectroscopy (830 nm), Baker et al. identified calcified material, buried within chicken breast and fatty tissues as well as normal and cancerous human breast tissues, at depths of 0.96 mm [131]. Matousek et al. used transmission Raman spectroscopy (827 nm excitation source) and successfully recovered Raman signal from calcified material buried within 16-mm-thick chicken breast tissue slabs [132]. Keller et al. in 2009 adopted this approach to look at the feasibility of spatially offset Raman spectroscopy (SORS) to determine cancer margins under a layer of normal breast tissue. They were able to detect breast cancer spectral signatures of tumors as small as 1–2 mm thick beneath up to 2 mm thick normal breast tissue [133]. Later, they improved sensitivity and specificity of this approach to 95 and 100%, respectively [134]. One potential problem with this approach is the differentiation of type II microcalcifications as benign or malignant using carbonate content. The ratio of phosphate and carbonate Raman bands at 960 and 1070 cm^−1^ helps to determine carbonate content. However, in subsurface Raman spectral analysis, Raman band at 1070 cm^−1^ is relatively weak and is overlapped with Raman collagen bands from the tissue. To overcome this problem, Kerssens et al. in 2010 came up with an alternate method. They realized that carbonate ion substitution leads to a perturbation of the hydroxyapatite lattice, which in turn affects the phosphate vibrational modes. By directly monitoring the position and bandwidth of the intense 960-cm^−1^ phosphate Raman band alone, they were able to determine carbonate content and thus differentiate type II microcalcifications as either benign or malignant to a depth of 5.6 mm using an 830-nm excitation source [135]. This approach is promising for noninvasive breast cancer screening.

Kneipp et al. looked at normal breast duct epithelial secretions using Raman spectroscopy to identify molecular changes that may occur during precancerous or cancerous conditions. They performed Raman spectroscopy using an 840-nm excitation source on breast secretions that cover the epithelium in most samples. The resulting spectra used PCA and *K*-means. The spectral signals from secretions were dominated by contributions from lipids. They observed two different classes of lipid secretion spectra that were sometimes identified on the same sample [136].

Yu et al. used micro-Raman spectra to compare normal and malignant human breast tissues. The observed spectral changes in cancerous tissue suggested decreased lipid content, increased nucleic acid content with conformation changes, and increased protein content with conformation changes and structural disorders such as broken molecular hydrogen bonds [137]. These results were corroborated by Yan et al. in 2005 and Yu et al. in 2006 using breast cancer cell lines [138, 139]. Yan et al. analyzed Raman spectra obtained using a 780-nm excitation source of normal and cancerous breast cells and reported decreased intensity of DNA phosphate groups and deoxyribose-phosphate in the cancer cells suggesting partial destruction of phosphate backbone [138]. Yu et al. looked at normal and transformed human breast epithelial cell lines using a 785-nm excitation source and reported that DNA duplication activities in tumorigenic cell nuclei are significantly higher than in normal cells [139].

Pichardo-Molina et al. in 2007, using an excitation source of 830 nm, looked at serum samples from 12 healthy volunteers and 11 patients that were clinically diagnosed with breast cancer using Raman spectroscopy. Using PCA and LDA, they detected significant spectral changes relating to proteins, phospholipids, and polysaccharides suggesting that this could be a potential approach for breast cancer detection [140].

Kast et al. in 2007 looked at mice normal and cancerous breast tissues from 17 mouse samples using Raman spectroscopy (785 nm excitation source). The lipid signatures were dominant in the normal mammary gland and associated lymph nodes, whereas the cancerous mammary glands showed increased protein and decreased lipid content. Inflamed mastitis tissue lacked the phospholipid peak at 1747 cm^−1^ and showed superimposed peaks in 1200–1500 cm^−1^. Furthermore, Raman spectral changes were detectable in preneoplastic changes in breast tissue [141].

Brozek-Pluska et al. in 2012 examined patient-matched normal and cancerous breast tissue using Raman spectroscopy with a 532-nm excitation source. They reported that regions characteristic for the vibrations of carotenoids, lipids, and proteins are the most important feature for differentiating normal breast tissue from cancerous breast tissue [142].

Abramczyk et al. in 2011 studied cancerous and noncancerous breast tissue from 146 patients using Raman spectroscopy (532 nm excitation source). They observed that lipid and carotenoids and fatty acid composition of cancerous breast tissue is markedly different from surrounding noncancerous breast tissue. The cancerous breast tissue was rich with metabolic products of arachidonic acid, whereas noncancerous breast tissue was rich with monounsaturated oleic acid and its derivatives [143]. They observed similar results in lipid droplets in breast cancer cells MCF-10A, MCF-7, and MDA-MB-231. The aggressiveness of cancer appeared to positively correlate with the amount of lipid droplets [144].

Surmacki et al. in 2013 studied normal and cancerous breast tissue from 200 patients using Raman spectroscopy (513 nm excitation source). They reported that specific protein–lipid–carotenoid profile and cell hydration are factors in the differentiation of cancerous and noncancerous breast tissue. The noncancerous breast tissue is rich with triglycerides (from adipose tissue) and fatty acids (from cell and nuclear membrane). The cancerous tissue was rich with protein content and had a greater amount of water [145].

#### Raman spectroscopy of breast cancer cell lines

Matthews et al. in 2011 used single-cell Raman spectroscopy (785 nm excitation source) to examine the effect of radiation on breast cancer cells MDA-MB-231 and MCF-7. PCA analysis of the Raman spectra identified radiation-induced biomolecular changes at the single-cell level independent of spectral variability arising from simultaneous processes such as cell cycle or cell death [146].

Marro et al. in 2018 studied metabolomic changes required for metastasis of triple negative breast cancer cell lines, MDA-MB-231 and MDA-MB-435. Raman spectra (532 nm excitation source) were analyzed using a multivariate curve resolution (MCR) method. It was determined that increased levels of tropism were associated with amino acids and lower levels of mitochondrial signals, whereas in lung tropism, both lipid and mitochondria (cytochrome c and RNA) levels are elevated [147].

Sialylation of glycolipids and glycoproteins on cancer cell surfaces (hypersialylation) is correlated with tumor metastaticity. Shashni et al. in 2017 used SERS to study hypersialylation of tumors with the aim of early detection of metastatic cancers. Phenylboronic acid-installed PEGylated gold nanoparticles was coupled with Toluidine blue O (T/BA-GNP) as SERS probe and measured surface sialic acid in metastatic cancer cell lines (MDA-MB-231) and on nonmetastatic cancer cell line (MCF-7). Strong SERS signals from metastatic cancer cell lines were observed, contrary to nonmetastatic cell lines. The detected SERS signals from various cancer cell lines correlated with their reported metastatic potential, implying that their SERS system can distinguish the metastaticity of cells based on the surface Neu5Ac density. The T/BA-GNP–based SERS system could also significantly differentiate between hypersialylated tumor tissues and healthy tissues with high SERS signal-to-noise ratio, due to plasmon coupling between the specifically aggregated functionalized GNPs [148].

Bi et al. in 2014 used Raman spectroscopy to study the human epidermal growth factor receptor 2 (HER2) amplification status and acquisition of drug resistance in breast cancer cells. HER2 overexpression is associated with increased breast cancer recurrence and worse prognosis. Lapatinib, the tyrosine kinase inhibitor, blocks HER2 signaling, but its activity is limited due to acquired drug resistance. The authors studied HER2 amplification and drug resistance of lapatinib using Raman spectroscopy in BT474 (HER2+ breast cancer cell), MCF-10A (HER2− control), and HER2+ MCF-10A (HER2+ control) cell lines. With 99% sensitivity and specificity, the authors observed HER2 overexpression. In Her2+ cells, lipid content was enhanced, and proteome was decreased. Lapatinib-resistant breast cancer cells retained lipogenesis even after lapatinib treatment [149].

Manciu et al. in 2016 used Raman spectroscopy (532 nm excitation source) to evaluate the biological activity of epidermal growth factor receptors on the surface of breast cancer cells with the goal of diagnosing breast cancer using specific receptor activity. Human epidermal growth factor receptor 1 (EGFR) overexpression is associated with cancer proliferation, and cancer treatments that are centered in targeting EGFR for therapy have shown to be effective. EGFR is present in very low concentrations making it difficult to detect using Raman. To overcome this problem, many tag EGFR with metallic nanoparticles and use SERS for detection. However, the authors wanted to develop a label-free method to observe changes in EFGR in nontumorigenic MCF-10A and tumorigenic MCF-7 breast epithelial cells using confocal Raman spectroscopy. They reported successful identification of EGFR using distinct Raman profiles relating to dominant changes in protein content and DNA/RNA characteristics. EGF addition resulted in modifications in lipid pool and DNA/RNA and vibrations from phosphorylated threonine and serine suggesting phosphorylation of signaling molecules upon addition of EFG to MCF-7 cells. This was confirmed by gel electrophoresis [150].

Hedegaard et al. in 2010 looked at two isogenic breast cancer cell lines (M-4A4 and NM-2C5) derived from the MDA-MB-435 cell line. Both are equally carcinogenic but M-4A4 is metastatic and NM-2C5 is nonmetastatic. Using Raman spectroscopy (785 nm excitation source), the authors observed that the metastatic cell line had polyunsaturated fatty acid content [151].

Abramczyk et al. in 2016 looked at MCF-10A, MCF-7, and MDA-MB-231 cells using Raman spectroscopy (532 nm excitation source). They specifically targeted epigenetic modifications—acetylation or methylation of lysine in cell nucleoli within the nucleus and lipid droplets in the cytoplasm. They reported overall increase in histone acetylation in the nucleoli of the cells with increase in aggressiveness of epithelial breast cancer cells. They observed that the stretching vibration of the methyl group blue-shifted in cancer cells from 2933 cm^−1^ for nonmalignant cells of MCF-10A to 2936 cm^−1^ for mildly malignant cells of MCF-7 and 2939 cm^−1^ for the aggressively malignant cells of MDA-MB-231 [152].

Medeiros et al. in 2016 studied the impact of dietary antioxidant isoflavone daidzein (DAID) on human breast cancer cells, MCF-7 (estrogen-dependent, ER+) and MDA-MB-231 (estrogen-independent, ER−), using Raman spectroscopy (785 nm excitation source). DAID is the second most abundant component of soybean and exhibits structural and functional similarities to the endogenous hormone estrogen. DAID may compete with natural estrogen in estrogen-dependent (ER+) breast cancers and, along with its high antioxidant property, could inhibit cancer cell growth or trigger cell death. Both MCF-7 and MDA-MB-231 cells exhibited a decrease in cell growth and proliferation in a dose-dependent manner. Its effect varied between estrogen-dependent and estrogen-independent cells. In MDA-MB-231 cells, cellular protein content was affected, and in MCF-7 cells, DNA and lipids were affected compared to control cells [153].

Mignolet et al. in 2017 studied differential effects of four polyphenols (epigallocatechin gallate (EGCG), gallic acid, resveratrol, and tannic acid) on MCF-7 breast cancer cells. Using a 532-nm excitation source, they obtained Raman spectra from each polyphenol-treated (for 24 h) MCF-7 cell. The spectra revealed that all four treatments led to increased lipid accumulation. Furthermore, there was an increase in cytochrome c into the cytosol in EGCG-treated cells that implies caspase activation and onset of apoptotic progress [154].

### Circulating tumor cell

Although the majority of cancer deaths result from cancer metastasis in a localized area (tumor), there is another important focus in current cancer research. Once a tumor reaches a stable size and growth, some cells separate and enter the bloodstream of the patient. These cells are referred to as circulating tumor cells (CTCs) [155–157]. Initial research in 1869 into cancer discovered not only the existence of CTCs but a possible relationship between CTCs and metastasized cancer [157]. Through research, CTCs have indicated information about cancer type, cancer progression, and patient response before, during, and after treatment [156, 158, 159]. During diagnosis, CTCs can assist in locating a cancer tumor, by indicating cancer type. This is done by growing a new tumor in a xenograft and identifying the cancer [156]. While the concentration of CTCs does not appear to reflect the actual size of an existing tumor, the presence of CTCs is a viable independent prognostic indicator for several cancers, including breast, prostate, and colon [157, 160, 161]. Additionally, regardless of initial levels, changes in concentration of CTCs in the patient do correspond to changes in the cancer tumor throughout treatment including re-occurrence after treatment is concluded [156].

There are several types and categories in CTC research; however, two categories have received attention beyond general CTC research. Cancer stem cells (CSCs) are a specific type of CTC with high metastatic activity, motility, and resistance to apoptosis. While CTCs can originate from benign tumors and are thus not necessarily pathogenic, CSCs are considered more likely to metastatic [156, 162]. Another topic of research is circulating tumor microemboli (CTM). CTM are multicellular aggregates of epithelial-like tumor cells and may also contain information about their tumor of origin [163].

There are currently three main detection research paths for CTCs: antibody capture, using cancer-derived DNA, and cytopathology. Each of these approaches has limitations that interfere with using the wealth of information CTCs may be able to provide. The most common current method in use with patients uses antibody capture based on epithelial marker epithelial cell adhesion molecule (EpCAM) on the CTCs. This method has an underlying assumption that is currently debated: CTCs have not undergone epithelial–mesenchymal transition [156, 157]. If this assumption is false, then not only will the result significantly underestimate the population of CTCs but miss a subpopulation of CTCs completely. Furthermore, certain cancers like carcinoma show partial mesenchymal properties. These properties appear to increase a cell’s metastatic potential, suggesting a greater correlation with the aspects of the tumor more relevant to treating the patient [156].

Another current method of detection involves isolating cancer-driven DNA in the plasma of the patient. This has the advantage of not requiring whole cells which would include CSCs and CTM. Various gene families including cytokeratins, prostate-specific antigens, and others studied through PCR showed correlation to metastasized cancer. While this method has its advantages, there are still too many issues with specificity and sensitivity of the results to use routinely [157].

The third method is not currently in use, but a proposed method. This approach relies on cytopathology, which is already used in screenings for other cancers such as PAP smears. Although this method innately has a higher specificity, due to the low concentration of CTCs in samples, the lack of sensitivity makes this approach impractical. Enrichment methods such as density gradient separation and filtration were unsuccessful in increasing the sensitivity of the tests because the multiple steps damaged or degraded the cells resulting in a loss of sensitivity and specificity [163].

Raman spectroscopy could provide increased specificity and sensitivity compared to the techniques described above. There have been three studies on applying Raman spectroscopy, mostly SERS, to CTC research. In 2008, SERS successfully detected CTC resulting from breast cancer using the same epithelial markers commonly used in CTC detection. Due to the specificity of SERS, the detection limit was 10 cells/ml with 99.7% confidence in buffer solution. This process had the advantage of needing very little sample preparation. Although there was no follow-up done with patients, this experiment provided proof of concept for Raman spectroscopy and CTCs [164, 165].

The next Raman experiment involved spiked blood serum. Microscopic Raman spectroscopy identified MCF-7 (breast cancer), BT-20 (breast cancer), OCI-AML3 (acute myeloid leukemia), leukocytes, and erythrocytes in suspension to mimic a clinical test. The results showed a prediction accuracy of 92.4% with a false positive less than 0.5%. This compares to the false positive of the cytopathology above of 1–3% [166].

Wang et al., using epidermal growth factor (EGF), detected CTCs at a concentration 50 tumor cells/ml of blood by detecting the expression of epidermal growth factor expression (EGFR) using SERS. Positive results for Tu212 SCCHN cells and H292 lung cancer cells (high EGFR expression), DA-MB-231 breast cancer cells (moderate EGFR expression), and H460 lung cancer cells (low EGFR expression) showed that even with a variation in expression, the system detected the cell’s existence. Further testing on 19 cancer patients with confirmed SCCHN showed that 17 out of 19 patients had CTCs (confirmed by filtration). Later, it was confirmed that the remaining two patients had localized instead of metastatic disease. Three control cancer-free patients showed no CTCs in the SERS. One research participant with a confirmed tumor was tested prior and following treatment. In this case, SERS appropriately indicated the presence of CTCs before treatment and their absence following treatment [165, 167]. Although this is a small sample size, the research indicates that SERS is a viable detection method for CTCs. Furthermore, this process has the additional advantage of not depending on epithelial cell markers. In addition to its increased accuracy, this approach may be used to detect CSCs or other CTCs that have undergone epithelial–mesenchymal transition.

### Other cancer

#### Raman spectroscopy of ocular tissue

On its way to the retina, light must travel through several transparent components [168]. The transparent properties provide a great opportunity for light-based detection techniques, including noninvasive use of Raman spectroscopy [168]. In ophthalmology, accurate readings of intraocular drug concentrations aid ophthalmologists in delivering optimal amounts of drugs in a patient’s eyes [169]. An important aspect of ophthalmology is ocular pharmacokinetic studies, which examine how drugs interact with the eye, such as how fast they metabolize and how quickly it diffuses over time [170]. These studies rely heavily on animal models, and the invasive techniques normally employed only allow for a single test subject to be used per time point [170]. As a result, ocular pharmacokinetic studies require many animals to serve as test subjects to properly assess an ophthalmic drug [170]. Due to its noninvasive nature, Raman spectroscopy could have significant advantage in studies and office use.

Bauer et al. in 1999 used laser scanning confocal Raman spectroscopy (LSCRS) to perform noninvasive pharmacokinetic assessments in live rabbits. They applied 25 μl of Trusopt 2%™ (a topical ocular drug) and measured changes in its signal amplitude over time in the tear film and corneal epithelium of six rabbits, successfully demonstrating the potential of Raman spectroscopy in ocular pharmacokinetics. They also speculated on the possibility of assessing drug–tissue interactions using resonance Raman spectroscopy and that drug-induced metabolic activity could be identified in tissue [170].

### Oral cancer

Oral cancer is a global issue with as many as 275,000 new cases arising each year [171]. The ability to diagnose oral cancer the first time it occurs and when it relapses has a direct impact on the 5-year survival rate, which currently stands at about 50% [172]. The prevalence of oral cancer on a national scale illustrates that it is more common in men than in women, most likely due to tobacco use habits [171]. In the USA and Europe, around 50% of oral cancers affect the tongue, though the floor of the mouth, gingivae, and palate are also sites for tumor growth [171]. For many patients, early diagnosis is the key to survival. Current methods of diagnosis often fail to detect precancerous and cancerous lesions at early stages [172]. These diagnoses of oral cancers are typically performed using a biopsy and histopathology of tissue, which can often be invasive. Another issue is this method relies on visual inspection, something found to only be useful in situations where a patient is at higher risk [172].

Barroso et al. examined specimens removed during a tongue resection on 14 patients with oral squamous cell carcinoma and determined their water content with high-wavenumber Raman spectroscopy. Measuring values from OH-stretching vibrations (3350–3550 cm^−1^) and CH-stretching vibrations (2910–2965 cm^−1^), they found that squamous cell carcinoma had significantly higher water content than the normal tissue nearby [173].

Singh et al. recorded *in vivo* Raman spectra using a high efficiency spectrograph and an excitation wavelength of 785 nm on 50 subjects with buccal mucosa. They took 215 spectra of normal tissue and 225 spectra of cancerous tissue. Analyzing in the region between 1200 and 1800 cm^−1^, they were able to achieve ~ 90–95% prediction efficiency with a model they created from the spectra [174].

## Conclusion and future direction

Raman spectroscopy can assist in uncovering the molecular basis of disease and provide objective, quantifiable molecular information for diagnosis and treatment evaluation. Numerous experimental studies have shown the capability of Raman spectroscopy for tissue characterization. The translation for clinical use involves the development of comprehensive spectral databases and tissue classification methodologies that can be compared with current gold standards. Best-practice techniques for data processing, acquisition, and classification need to be developed and adopted. Various interferents, such as fluorescence, a process that usually “competes” with Raman scattering, can hamper the interpretation of Raman spectra of biological samples. Preprocessing the raw data helps eliminate unwanted signals, enhances Raman spectral features, and allows more reproducible data for qualitative and quantitative analysis. However, it has been demonstrated by us and others that the choice of preprocessing strategy can greatly influence tissue classification results. In addition to developing best-practice techniques for spectral preprocessing, care must be taken when developing classification algorithms for diagnostic evaluation. Validation studies need to be performed to confirm that algorithms developed on *ex vivo* specimens are applicable to *in vivo* tissues. Machine learning algorithms hold the promise of automating the identification and diagnosis of cancer. Deep learning training, using large numbers of spectra, may also identify molecular patterns among cancer types, aid in margin detection, and become predictors of the aggressiveness of the cancer.

In addition to algorithm development, laser tissue interactions that might result in tissue damage need to be investigated to translate the technology to clinical application. Raman scattering strength is proportional to the inverse of excitation wavelength to the fourth power and proportional to intensity of the incident light. Even though more light translates to more signal, tissue has a damage threshold.

The continued development of Raman spectral databases, tissue classification methodologies, and instrument designs trending toward obtaining data with greater resolution, shorter collection times, and higher accuracy will ensure that Raman spectroscopy becomes a powerful tool in clinical application.
